# Classification of pediatric acute myeloid leukemia based on miRNA expression profiles

**DOI:** 10.18632/oncotarget.16525

**Published:** 2017-03-23

**Authors:** Askar Obulkasim, Jenny E. Katsman-Kuipers, Lonneke Verboon, Mathijs Sanders, Ivo Touw, Mojca Jongen-Lavrencic, Rob Pieters, Jan-Henning Klusmann, C. Michel Zwaan, Marry M. van den Heuvel-Eibrink, Maarten Fornerod

**Affiliations:** ^1^ Pediatric Oncology-Hematology, Erasmus MC, Sophia Children's Hospital, The Netherlands; ^2^ Department of Hematology, ErasmusMC, Rotterdam, The Netherlands; ^3^ Prinses Máxima Center for Pediatric Oncology, Utrecht, The Netherlands; ^4^ Department of Pediatric Hematology and Oncology, Hannover Medical School, Hannover, Germany

**Keywords:** microRNA, acute myeloid leukemia, pediatric, classification, cytogenetic aberration

## Abstract

Pediatric acute myeloid leukemia (AML) is a heterogeneous disease with respect to biology as well as outcome. In this study, we investigated whether known biological subgroups of pediatric AML are reflected by a common microRNA (miRNA) expression pattern. We assayed 665 miRNAs on 165 pediatric AML samples. First, unsupervised clustering was performed to identify patient clusters with common miRNA expression profiles. Our analysis unraveled 14 clusters, seven of which had a known (cyto-)genetic denominator. Finally, a robust classifier was constructed to discriminate six molecular aberration groups: 11q23-rearrangements, t(8;21)(q22;q22), inv(16)(p13q22), t(15;17) (q21;q22), NPM1 and CEBPA mutations. The classifier achieved accuracies of 89%, 95%, 95%, 98%, 91% and 96%, respectively. Although lower sensitivities were obtained for the *NPM1* and *CEBPA* (32% and 66%), relatively high sensitivities (84%−94%) were attained for the rest. Specificity was high in all groups (87%−100%). Due to a robust double-loop cross validation procedure employed, the classifier only employed 47 miRNAs to achieve the aforementioned accuracies. To validate the 47 miRNA signatures, we applied them to a publicly available adult AML dataset. Albeit partial overlap of the array platforms and molecular differences between pediatric and adult AML, the signatures performed reasonably well. This corroborates our claim that the identified miRNA signatures are not dominated by sample size bias in the pediatric AML dataset. In conclusion, cytogenetic subtypes of pediatric AML have distinct miRNA expression patterns. Reproducibility of the miRNA signatures in adult dataset suggests that the respective aberrations have a similar biology both in pediatric and adult AML.

## INTRODUCTION

Currently, pediatric AML patients are stratified into risk categories according to response to induction therapy and genetic abnormalities, as defined in the WHO 2008 classification [1]. Although patient outcome has improved over the past decades, overall survival rates are 60–70% and the relapse rate is still high [2–4]. To further improve patient outcome, biological studies that aim to identify leukemogenic drivers and/or signaling pathways that can be directly targeted are needed. This is necessary as further intensification of chemotherapy may cause higher frequency of early and late side effects, including therapy-related mortality [5]. Alternatively, patient outcome may be improved by refining the risk-group classification and identify uniform subgroups utilizing (epi-) genetic and molecular aberrations, which may also contribute to the design of targeted therapy [6]. Identification of hallmark aberrations and their accompanying molecular targets for the 15–20% of unclassified pediatric AML patients is the subject of many ongoing biological studies [7].

Mutations in AML can be categorized into Type I and Type II classes [42]. Type I aberrations occur in genes involved in cell proliferation and survival (e.g., *FLT3*, *KIT*, *NRAS* and and *TP53*) while Type II aberrations lead to impaired differentiation. Examples of these are PML-RARα, *MLL*-rearrangements and CBFB-MYH11.

MiRNAs - circa 22 nucleotide long non-coding RNAs - influence gene expression by suppressing the translation of genes that have sequences complementary to the miRNA in their 3′UTR. Since a miRNA can target multiple genes, it therefore can influence many physiological processes like apoptosis, proliferation, differentiation, and ageing as well as hematopoietic differentiation [8–13]. The epigenetic effect of miRNA on gene expression contributes to leukemogenesis through interaction with tumor suppressor genes that are involved in cell proliferation and differentiation [10, 14–17]. Although cancer-promoting miRNAs have been described in adult AML, there are unmet demands for studies on role of miRNA in pediatric AML [18–21]. Zhang *et al*. described miR-expression differences between FAB-M1, FAB-M2 and FAB-M3 groups in pediatric AML [21]. In our previous work, we demonstrated the non-random distribution of miR-29a, miR-155, and miR-196a/b expressions between clinically relevant genetic entities of pediatric AML [18]. Daschkey *et al*. performed clustering on pediatric samples with t(8;21), t(15;17) and *MLL*-rearrangements using their miR-expression profiles [19]. We also showed that inv(16) and other genetic aberrations in pediatric AML have specific miR-expression profiles. Furthermore, low expression of the miR-9 was identified to act as a tumor-suppressor in cooperation with let-7 family members in a stringent cell-context dependent manner in pediatric AML samples with t(8;21) [20]. In another it has been identified that high expression of the miR-99a, miR-125b and let-7c in pediatric AML with FAB M7 phenotype promoted leukemogenesis by switching the balance between TGFß and Wnt signaling [22]. However, so far, no study has been conducted to identify novel prognostic subgroups of pediatric AML based on the miRNA profiles. To address this, in the work we investigated, in a large cohort, whether genetic and molecular subtypes of childhood AML can be classified using their miRNA expression profiles.

## RESULTS

### Unsupervised clustering

**Figure 1:**
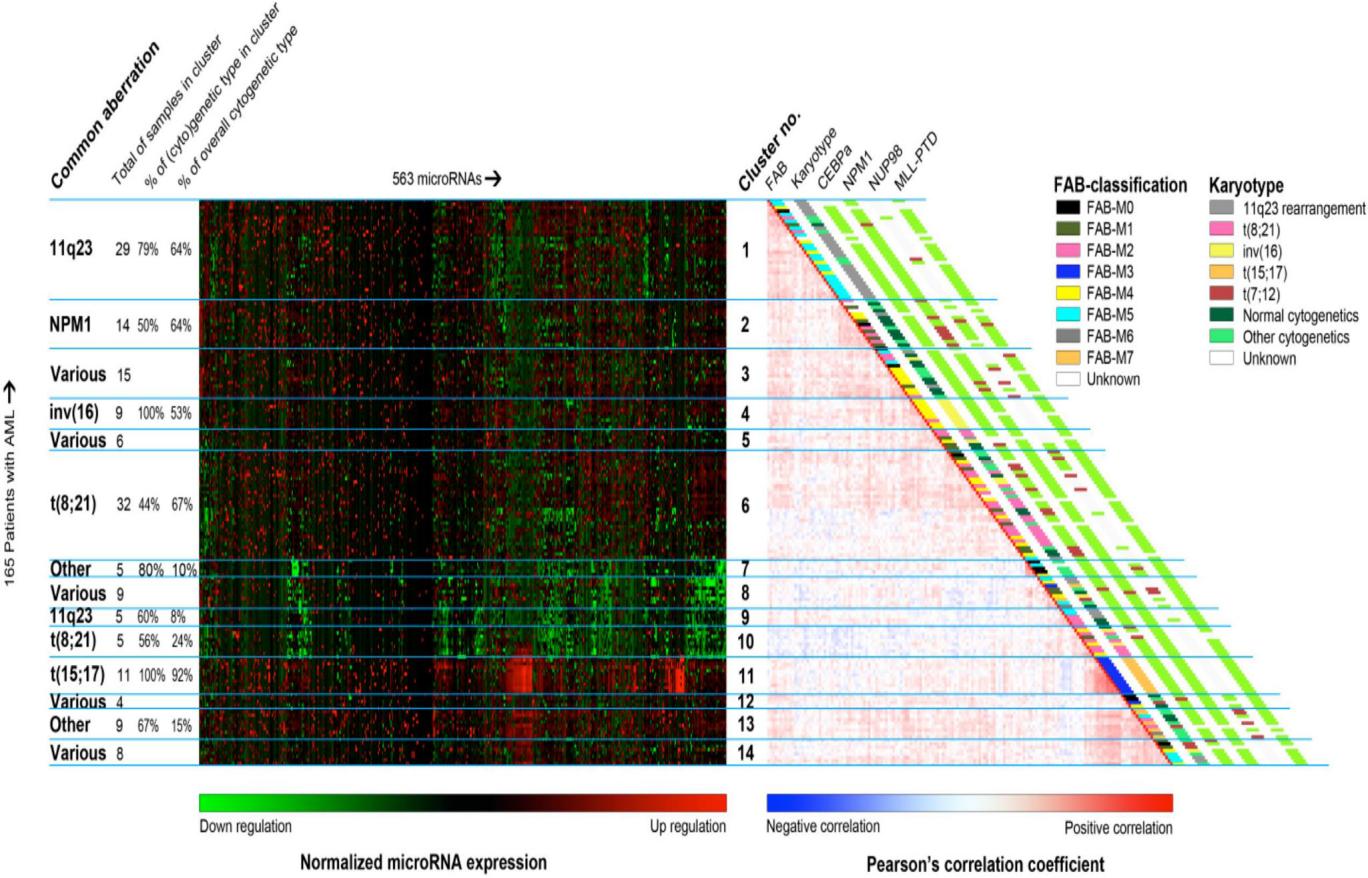
Unsupervised clustering of the pediatric AML samples using their miRNA profiles This combination of heatmaps shows the miRNA expression profiles of 165 pediatric AML patients divided 14 clusters found through semi-unsupervised clustering based on miRNA expression of 563 miRs. The selected miRNAs provided the clearest division between clusters. Left-hand side: normalized miRNA expression values with green meaning down regulation and red up regulation. The rows were ordered based on the correlation plot on the right. Columns were ordered by hierarchical clustering. Right-hand side: Pairwise correlation between 165 pediatric patients with acute myeloid leukemia. The cells are colored by Pearson's correlation coefficient values ranging from dark blue (negative correlation) to dark red (positive correlation). FAB-type, Karyotype, *CEBPA* mutation status, *NPM1* mutation status, *NUP98*-rearrangements and MLL-PTD are plotted alongside the graph. For the clinical characteristic that have no plotted legend, green means the characteristic is absent, red means that it is present and white means that the sample was not tested for this characteristic.

We began by performing hierarchical clustering of patients using a subset of miRNAs. The miRNAs were selected if they were 6-fold higher or lower expressed than that of the geometric mean of the cohort in at least one patient sample. This resulted in selection of 563 out of 664 arrayed miRNAs. The clustering was performed using Pearson correlation as distance metric and Ward's minimum variance as linkage. Clustering results are shown in Figure 1. The clusters subsequently were examined for the enrichment of cytogenetic and molecular aberrations, FAB-classification, and mean age and white blood cell count (Supplementary Table 1), separately. We observed that miRNAs expressions correlated with cytogenetic and molecular aberrations groups, especially with *MLL*-rearrangements, *NPM1* mutations, inv(16)(p13q22), t(8;21)(q22;q22), and t(15;17)(q21;q22). However, the clusters were not enriched for type I aberrations (FLT3-ITD, FLT3-TKD, and mutations in *WT1*, *NRAS*, *KRAS*, *PTPN11*, and *C-KIT*).

The samples with *MLL*-rearrangements appeared to be separated into two sub-clusters: cluster 1 and cluster 9. But, this separation was not related to the *MLL*-translocation partner. Majority of samples with *NPM1*-mutations were located adjacent to the samples with *MLL*-rearrangements in the cluster 2. Four samples with *NPM1* mutations were scattered in the cluster 1 (*n* = 1), cluster 3 (*n* = 2) and cluster 8 (*n* = 1).

Fifty-three percent of the samples (*n* = 17) with inv(16)(p13q22) were found in the cluster 4 and the rest were scattered over 6 clusters. The samples with t(8;21)(q22;q22) grouped closely together in the cluster 6 and the cluster 10, two cases were found in the heterogeneous cluster 5 that encompasses samples with various aberrations. We presumed that the distribution of samples with t(8;21)(q22;q22) in two sub-clusters might be due to differences in type I mutations or morphological features. However, no significant differences between these clusters were observed based on the afore-mentioned characteristics (Supplementary Table 2). All samples with *CEBPA* double mutations were clustered together with the t(8;21)(q22;q22) samples.

Eleven out of the twelve samples with t(15;17)(q21;q22) were present in the cluster 11. We observed no enriched biological characteristic, e.g. type I aberration, of the outlying sample.

The cluster 7 and 13 were consisted of > 65% of samples (*n* = 4 and *n* = 6) with “other” cytogenetics but no known common cytogenetic or molecular denominator was found.

For the three cytogenetic groups, i.e. the t(7;12)(q36;p13, t(8;16)(p11;p13), and NUP98-rearrangements, no homogeneous cluster was found.

### Classification of genetic and molecular subtypes

To investigate the potential of miRNAs in predicting known Type II aberration subtypes, we performed classification using samples with *MLL*-rearrangements, t(8;21)(q22;q22), inv(16)(p13q22), t(15;17)(q21;q22), *CEBPA* double mutations and *NPM1* mutations. Subtypes selection was purely due to control sample sizes. We generated miRNA signatures specific to each genetic subtype using Support Vector Machine (SVM). As previously described, a double-loop-cross-validation (DLCV) strategy avoids over-fitting and leads to stable signatures with highest prediction accuracy [24]. Details of about classification model construction steps are given in the Supplementary information.

The mean prediction accuracy of 100 double-loop cross-validation (DLCV) runs in the second loop is given in Table 1. We observed that the classifier rendered high prediction sensitivities in the subtypes of *MLL*-rearrangements, t(8;21)(q22;q22), inv(16)(p13q22) and t(15;17)(q21;q22), ranging from 84–94%. In the groups with *CEPBA* double mutations and *NPM1* mutations, however, low prediction sensitivities were observed (66% and 32%, respectively). The specificities in all subtypes were high (87–100%). The optimal set of 47 miRNAs signatures and their expression relative to the other subtypes are given in Supplementary Table 4. It can be seen that high expression of *miR-362-5p* and low expression of *miR-133a* is specific for samples with *MLL*-rearrangements, while high expression of *miR-369-3p*, *miR-369-5p*, *miR-409-5p*, *miR-485-5p*, *miR-654-3p*, and *miR-654-5p* is specific for samples with t(15;17).

**Table 1:** Classification results achieved on the pediatric AML dataset

(%)	Sensitivity	Specificity	PPV	NPV	Accuracy
**MLL(*n* = 36)**	93	87	79	97	89
**t(8;21)(q22;q22)(*n* = 21)**	94	95	84	99	95
**inv(16)(p13q22)(*n* = 17)**	84	97	86	97	95
**t(15;17)(q21;q22)(*n* = 12)**	85	100	97	98	98
**CEPBA.dm(*n* = 11)**	66	100	97	96	96
**NPM1(*n* = 11)**	32	98	46	93	91

Note: PPV- positive predictive value, NPV- negative predictive value.

Samples with *MLL*-rearrangements were classified with 89% accuracy using 37 miRNA expression signatures; 14 miRNAs were highly expressed and 23 were lowly expressed compared to the samples without *MLL*-rearrangements. Among the 23 downregulated miRNAs, seven are known tumor suppressors: *let-7f*, *miR-126*, *miR-181a*, *miR-181c*, *miR-195*, *miR-26a*, and *miR-29a*32. Two miRNAs were only used to classify *MLL*-rearranged samples: lowly expressed *miR-133* and highly expressed *miR-362-5p*. Classification of samples with *NPM1* mutations was achieved 91% accuracy by only using five miRNAs: *let-7c, let-7f*, and *miR-196b* were highly expressed and *miR-126* and *miR-320* lowly expressed. The samples carrying inv(16)(p13q22) were characterized by 17 miRs; three were lowly expressed, 14 were highly expressed.

Fourteen miRNAs were used to classify samples with t(8;21)(q22;q22). Among them, eight were lowly expressed and six were highly expressed. The 14 miRNA expression signature largely overlapped with the signature specific to inv(16) subtype. The classifier generated 18 miRNA signatures specific to the samples with *CEBPA* double-mutations that resulted in classification accuracy of 96%. Among them 12 were lowly expressed and six highly expressed miRNAs. The samples carrying t(15;17)(q21;q22) were classified with accuracy of 98% by 14 upregulated miRs, of which six miRs, *miR-369-3p*, *miR-369-5p*, *miR-409-5p*, *miR-485-5p*, *miR-654-3p*, and *miR-654-5p*, were specific to this group.

### Validation of the 47 miRNA signatures

To examine the validity of the 47 miRNA signatures generated on the pediatric AML dataset, we test their prediction power using an independent dataset. Due to lack of a sufficiently large pediatric AML dataset, we used an adult AML dataset from Jongen-Lavrencic et al [42] instead. The t(15;17)(q21;q22) group was excluded from the analysis due to insufficient samples. Classification results are given in Table 2. Relatively high classification sensitivities were obtained in the t(8;21)(q22;q22), inv(16)(p13q22), *CEBPA* double-mutations and *NPM1* mutations groups (83%–100%), whereas, the *MLL*-rearrangements displayed a lower sensitivity (50%). Similar to the pediatric dataset, high specificities were obtained in all groups (84%–100%).

**Table 2:** Validation of the 47 miRNA signatures, generated from the pediatric AML dataset, on the adult AML dataset

(%)	Sensitivity	Specificity	PPV	NPV	Accuracy
**MLL(*n* = 12)**	50	100	100	95	95
**t(8;21)(q22;q22)(*n* = 12)**	100	100	100	100	100
**inv(16)(p13q22)(*n* = 15)**	100	100	100	100	100
**CEPBA.dm(*n* = 16)**	83	100	100	97	98
**NPM1(*n* = 68)**	100	84	88	100	93

Note: PPV- positive predictive value, NPV- negative predictive value.

## DISCUSSION

Unsupervised clustering using expression of 665 human miRNAs revealed the discriminative power miRNA for samples with cytogenetic abnormalities, i.e. *MLL*-rearrangements, *NPM1* mutations, inv(16)(p13q22), t(8;21)(q22;q22), and t(15;17)(q21;q22). Furthermore, classification of selected cytogenetic groups using only 47 miRs resulted in high sensitivity. These results may reflect the relative homogeneous biology of these subtypes in terms of their miRNA profiles.

Our results are in line with previous study that reported strong miRNA signatures for pediatric AML carrying inv(16)(p13q22) and t(8;21)(q22;q22) [19]. Multiple studies on adult AML also reported predictive miRNA markers for t(15;17)(q21;q22) and the CBF-leukemias [33–35]. One of the clusters showed in our study enriched for sample with *NPM1* mutations (64%), indicating there is a *NPM1* mutation specific miRNA expression profile, which concordant with studies on adult AML [36]. Interestingly, although *NPM1* cases cluster together in unsupervised analysis, classification of these samples only resulted in sensitivity of 32%. This might be due to (a) the number of included samples; (b) cooperating genomic events that influence the miRNA profiles of the samples [43]. The *MLL*-rearranged specific miRNA signatures, however, were not previously reported either in pediatric or in adult AML.

To quantify the predictive power of miRNA expression in discriminating the cytogenetic subtypes in a supervised way, we performed classification using multiple well-known classification algorithms, which are given in the Supplementary Information. We observed that the classification accuracies are relatively uniform across algorithms. This corroborates our findings, and our original decision to use SVM as a classifier is free from selection bias.

Overall, classification accuracies using samples’ miRNA expression profiles were not exceeded that of the classification using gene expression profiles previously reported [24]. Superior performances of the mRNA-based classifier over the miRNA-based one have also been reported in two studies on adult AML [35, 37].

The power of our classifier might be further validated using an independent pediatric AML cohort. Unfortunately, currently datasets that can be used for validation are scarce. In this study, we were only able to validate out classifier with an adult AML dataset, which we think is the closest replication cohort [35–37].

Majority of the miRNAs reported here were overlapped with the ones mentioned by Jongen-Lavrencic *et al*: *miR-485-5p* for t(15;17)(q21;q22), miR-126*, miR-196b, and *miR-9* for t(8;21)(q22;q22), miR-126*, miR-30b, and *miR-335* for inv(16)(p13q22), *miR-149, miR-181a, miR-181c, miR-196b*, and *miR-9* for *CEBPA* double-mutated cases. In a study on adults AML it has been reported that miRNAs could be used for class prediction of t(15;17)(q21;q22), t(8;21)(q22;q22) and inv(16)(p13q22) [35]. Li *et al*. used 7 miRNAs for class prediction of CBF-leukemia (inv(16)(p13q22) and t(8;21)(q22;q22) together), t(15;17)(q21;q22) and *MLL*-rearrangements, which resulted in an accuracy of > 94% in a cohort of 52 adults using bead arrays [37]. We confirmed the potential of *miR-126*/miR-126* over expression in CBF leukemias.

*MiR-485-5p* was the only miRNA used to classify both adult and pediatric AML samples with t(15;17) [35, 37]. The *MiR-485-5p* is encoded on chromosome 14q32.31, and overexpression of several miRNAs located adjacent to each other on this location has been reported in adult. However, these miRNAs were not used for classifying the adult AML samples with t(15;17). Interestingly, the miRNA signatures specific to the t(15;17)(q21;q22) group are encoded on chromosome 14q32.31 [34]. The mechanism behind their overexpression and possible function in AML has not been elucidated yet. We believe findings might provide clues regarding the distinct biology of t(15;17)(q21;q22) positive AML.

Down regulation of *miR-126, miR-196b*, and *miR-9* was found in both pediatric and adult t(8;21)(q22;q22) AML. In our recent we showed that down regulated *miR-9* acts as a tumor suppressor in pediatric t(8;21)(q22;q22) AML and induced differentiation through targets HMGA2 and LIN28B in cooperation with the let-7 family. It might be worthwhile to explore the therapeutic potential by ectopic expression of *miR-9* in t(8;21)(q22;q22) positive AML [20].

The miR-126*, miR-30b, miR-30d, and *miR-335* were also used to classify adult AML samples with inv(16)(p13q22) [35, 37]. It has been reported there were relationship between down regulation of *miR-30b* and overexpression of oncogene *MYBL2*, and poor prognosis [38].

While the *miR-10a* and *miR-10b* were used to classify adult AML saples with *NPM1* mutations, neither of them was selected by our classifier when discriminating the samples with *NPM1* mutations in our cohort [36, 39]. This might reflect the differences in leukemogenic pathways between children and adults or may be merely due to the method of miRNA-selection. In addition, the miRNA profiles of *NPM1*-mutated samples resemble the profiles of *MLL*-rearranged samples, which explain why these samples ended up in the same cluster.

Expression of five miRNAs, *miR-149*, *miR-181a*, *miR-181c*, *miR-196b*, and *miR-9*, in pediatric AML samples with CEBPA mutations were also reported in adult AML case with *CEBPA* mutations [33, 35, 37, 40]. Over expressions of tumor suppressive *miR-181* family were found in *CEBPA*-mutated cases and has been showed to correlate with treatment response and better clinical outcome in AML patients. Treatment of AML-blasts carrying *CEBPA*-mutations with lenalidomide sensitized AML cells to chemotherapy and increased CEBPA-p30 protein levels and miR-181a expression [41].

To examine if the miRNA signatures given in Supplimentary Table 4 are characterized by distinct target gene patterns, thus reflecting disease biology, we performed the following analyses: we downloaded 289 miRNAs from five miRNA-mRNA target prediction databases: microcosm, mirecords, mirtarbase and pita targetscan. Among the 47 unique miRNAs given in Table 3, only 25 were found in the miRNA-mRNA target prediction databases we downloaded (see Supplementary Table 3). To reliably predict miRNA target genes, we call a gene as the target of the miRNA under investigation if the prediction is reported at least in three databases. It is known that a miRNA expression is inversely correlated with the expression of its targeted genes. To investigate if this prior knowledge holds in our dataset, we obtained mRNA expression dataset that were measured on the same samples24. Visualizations of the inverse correlation between miRNA and targeted genes were displayed in Supplementary Figure 1–58. While some genes behaved consistent with the prior assumption, i.e. inverse correlation, we also observed genes that violated the assumption. We believe this worth further investigation.

**Table 3:** The list of miRNA signatures that were arrayed on the adult AML dataset platform

t(8;21)(q22;q22)	inv(16)(p13q22)	t(15;17)(q21;q22)(*n* = 12)	CEPBA.dm	NPM1
miR-126*	miR-126*	miR-485-5P	miR-149	miR-196b
miR-196b	miR-30b		miR-181a	miR-320
miR-9	miR-30d		miR-181c	
	miR-335		miR-196b	
			miR-9	

To sum up, (cyto)genetic aberrations groups have specific miRNA expression profiles both in pediatric and adult. In unsupervised, some patients with unknown underlying common genetic or molecular denominator were clustered together. Further investigations focusing on the common factor in these clusters might reveal new subgroups of pediatric AML. Although relatively high classification results were obtained from the six aberration groups investigated in this study, they did not exceed the results from currently used methods, which limits its clinical applicability. We believe that the miRNAs signatures reported here spark future line of research as they may provide insights into important biological pathways involved in pediatric AML.

## MATERIALS AND METHODS

Viably frozen diagnostic bone marrow or peripheral blood samples from 165 *de novo* pediatric AML cases were provided by the Dutch Childhood Oncology Group, the AML ‘Berlin-Frankfurt-Münster’ Study Group, the Czech Pediatric Hematology Group and the St. Louis Hospital in Paris, France. Samples represented the most common and relevant cytogenetic groups and were selected based on the availability of high-quality RNA. Informed consent was obtained from all patients, after Institutional Review Board approval, according to national law and regulations. Samples were enriched to contain at least 80% leukemic cells as previously described [23]. Routine analysis of recurrent non-random cytogenetic aberrations was performed by standard chromosome banding analysis, RT-PCR, and split-signal FISH (*MLL*-rearrangements, inv(16)(p13q22), t(8;21)(q22;q22), t(15;17)(q21;q22), t(7;12)(q36;p13), t(8;16)(p11;p13), and *NUP98*-rearrangements) and hotspot mutations in genes (*NPM1*, *CEBPA*, *FLT3-ITD*, *NRAS*, *KRAS*, *PTPN11*, c-*KIT*, and *MLL-PTD*) as described previously [24–30].

MicroRNA expression profiling was performed by Taqman^®^ Array MicroRNA Cards v2.0 (Applied Biosystems, Foster City, CA, USA). Raw Ct-values were analyzed, summarized and exported using SDS 2.3 (Applied Biosystems). All further biostatistical analyses including quality control, aggregation of data, data normalization, and filtering were performed using R 2.11.1 [31].

Unsupervised hierarchical clustering of samples was performed using 563 miRNAs. These miRNAs were 6-fold higher or lower expressed compared to the geometric mean of the cohort in at least one patient sample (Pearson correlation and Ward distance) within the R 3.0.3 statistical environment.

A classifier was constructed for the groups identified by unsupervised clustering with at least ten samples. We used the classification strategy described in Balgobind *et al*. [24]. Normalized adult AML data [42] was obtained from the last author. Due to difference in data generating platforms, only part of the 47 miRNA signatures were found in the adult AML dataset (See Table 3). During the validation, a classifier was trained using the pediatric AML data with only those overlapped miRNA signatures. Then, the trained classifier was applied to the adult AML dataset to quantify the prediction accuracy.

## SUPPLEMENTARY MATERIALS FIGURES AND TABLES






