# Supplementary material for: Classification of pediatric acute myeloid leukemia based on miRNA expression profiles
Source: Oncotarget. 2017 Mar 23;8(20):33078–85. doi: 10.18632/oncotarget.16525 (PMC5464851; doi:10.18632/oncotarget.16525)
Supplement: Supplementary file 1 [file oncotarget-08-33078-s001.pdf]

Classification of pediatric acute myeloid leukemia based on miRNA expression profiles

Supplementary Materials

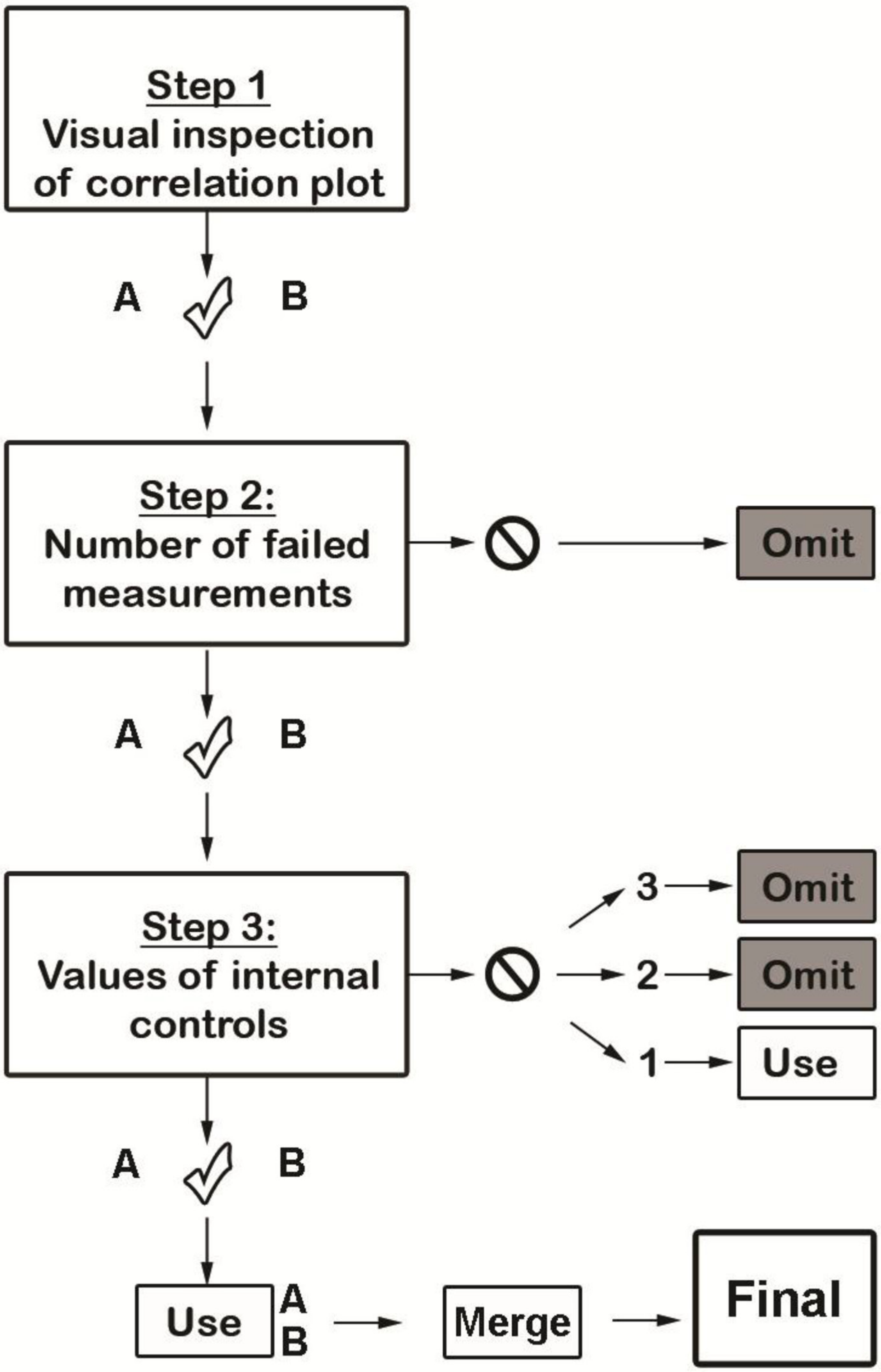

Supplementary Figure 1: Flowchart for large scale HT-qPCR quality control. Three-step flowchart for quality control of large scale HT-qPCR experiments as used in our pediatric AML cohort. Failed measurements: measurements are considered failed when flagged by the SDS-program or when the Ct values are higher than 38 or not acquired (NA).

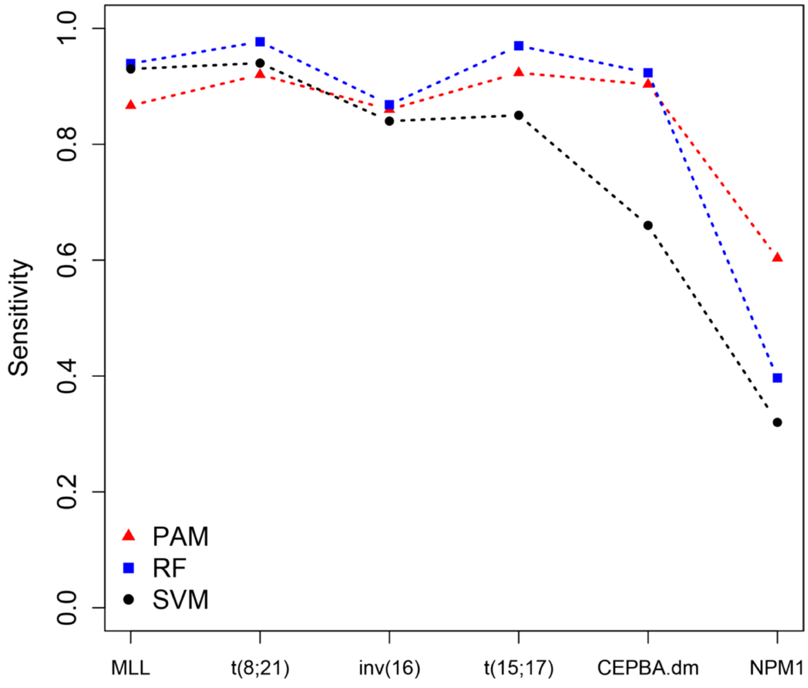

Supplementary Figure 2: Sensitivity of the classifiers. Classification performances from the three different classification algorithms on the miRNA data used in this study. The x-axis shows the cytogenetic subtypes, the y-axis the sensitive of the classifier.

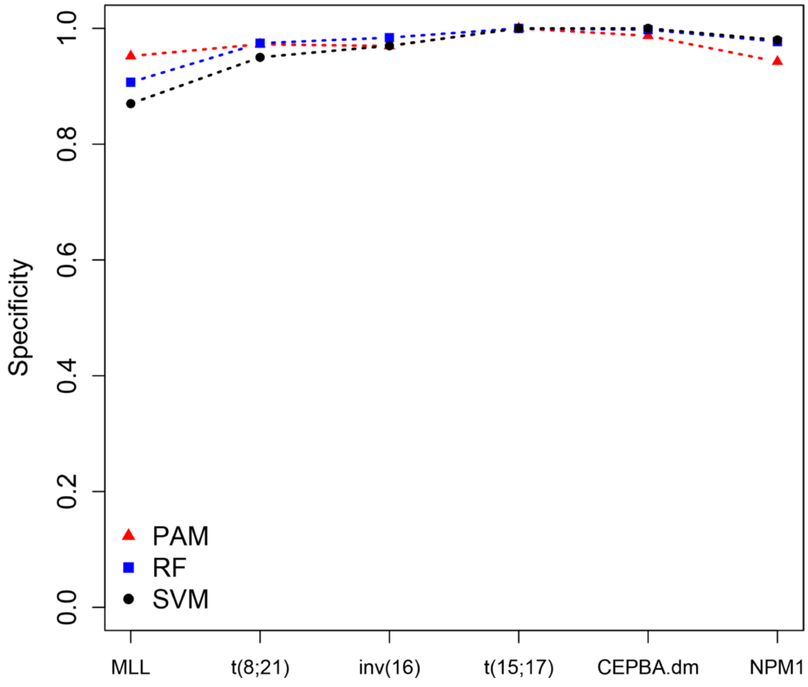

Supplementary Figure 3: Specificity of the classifiers. Classification performances from the three different classification algorithms on the miRNA data used in this study. The x-axis shows the cytogenetic subtypes, the y-axis the specificity of the classifier.

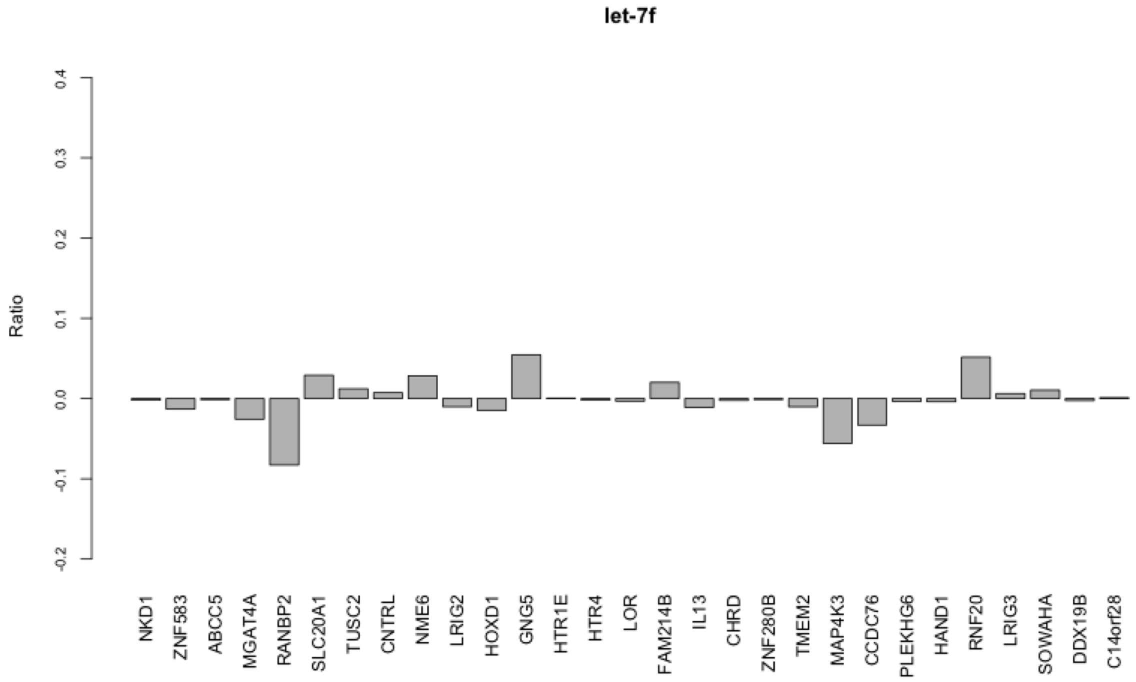

Supplementary Figure 4: Expression of the target genes of let-7f in the *MLL*-rearrangement groups compared to that of the rest.

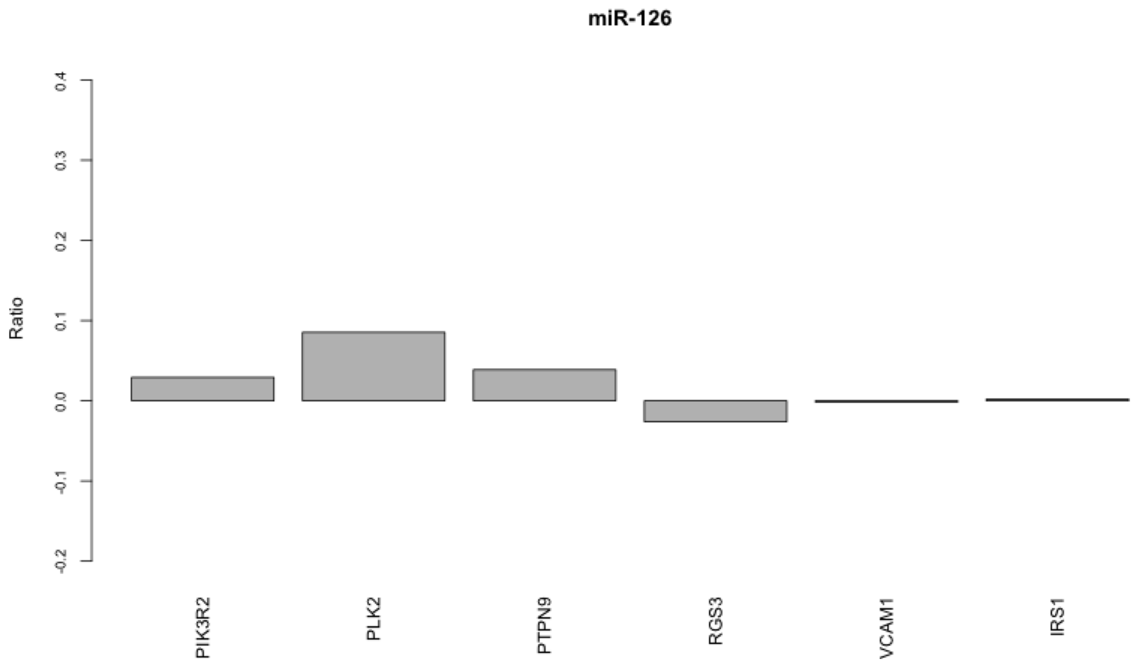

Supplementary Figure 5: Expression of the target genes of mirR-126 in the *MLL*-rearrangement groups compared to that of the rest.

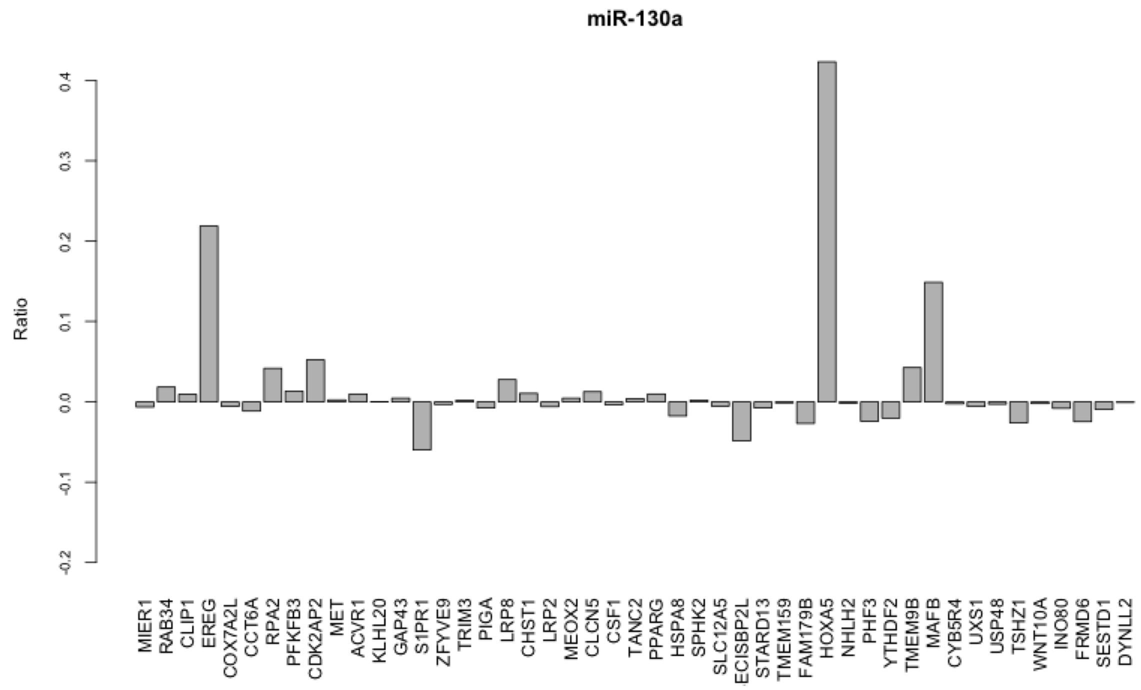

Supplementary Figure 6: Expression of the target genes of miR-130a in the *MLL*-rearrangement groups compared to that of the rest.

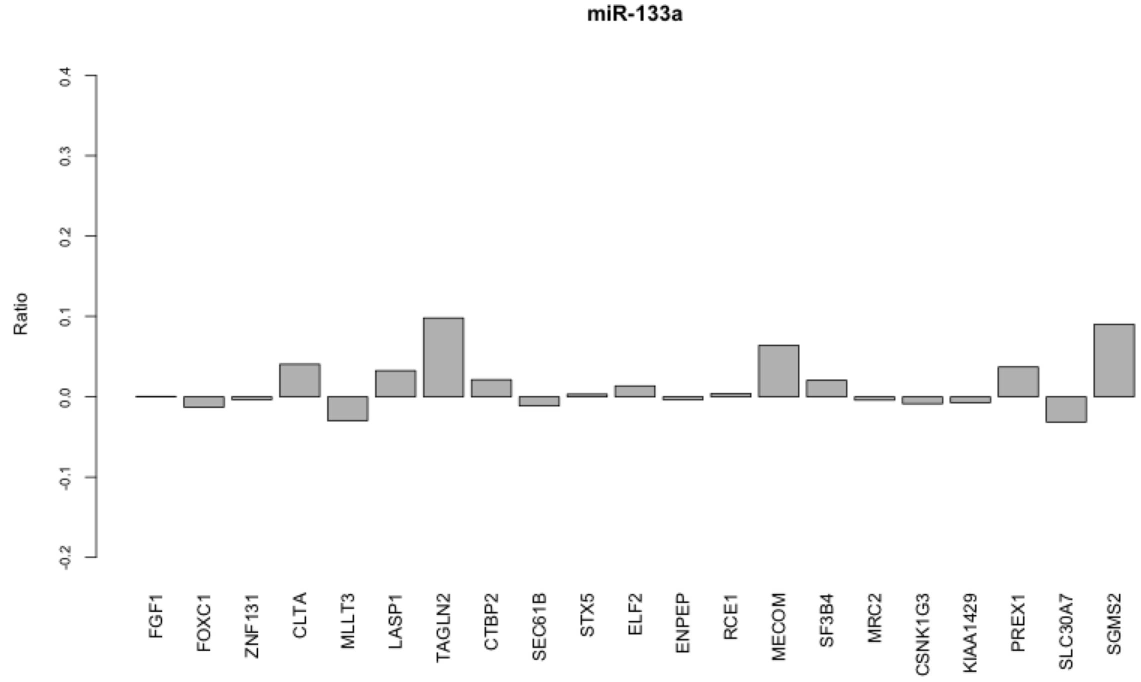

Supplementary Figure 7: Expression of the target genes of miR-133a in the *MLL*-rearrangement groups compared to that of the rest.

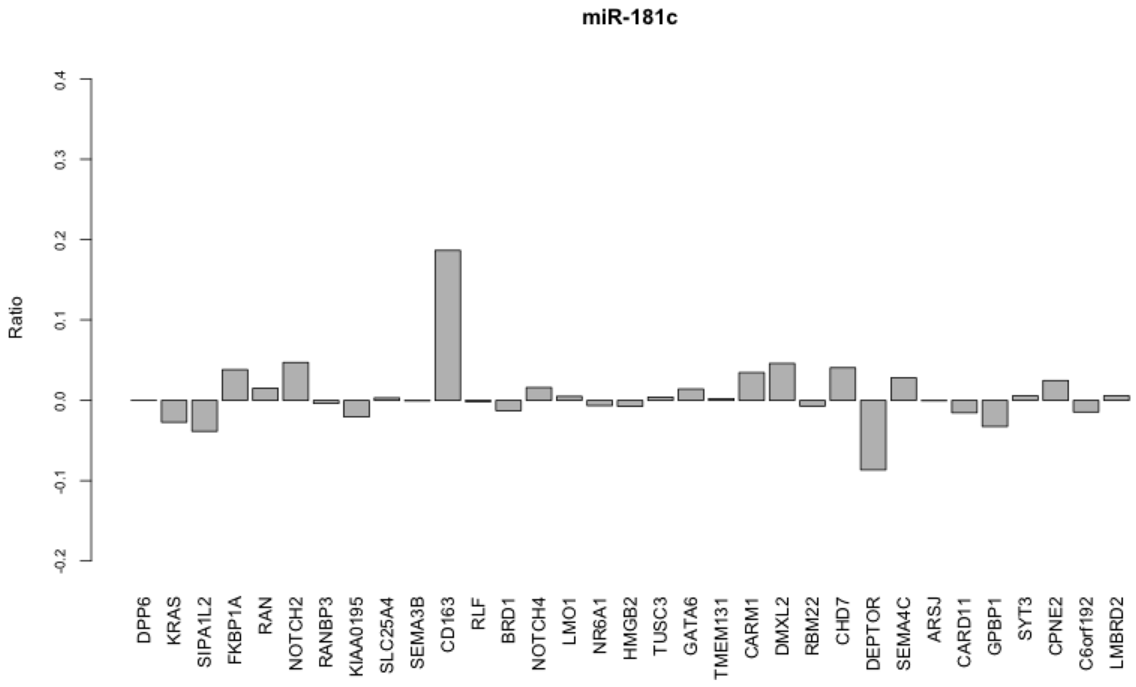

Supplementary Figure 8: Expression of the target genes of miR-181c in the *MLL*-rearrangement groups compared to that of the rest.

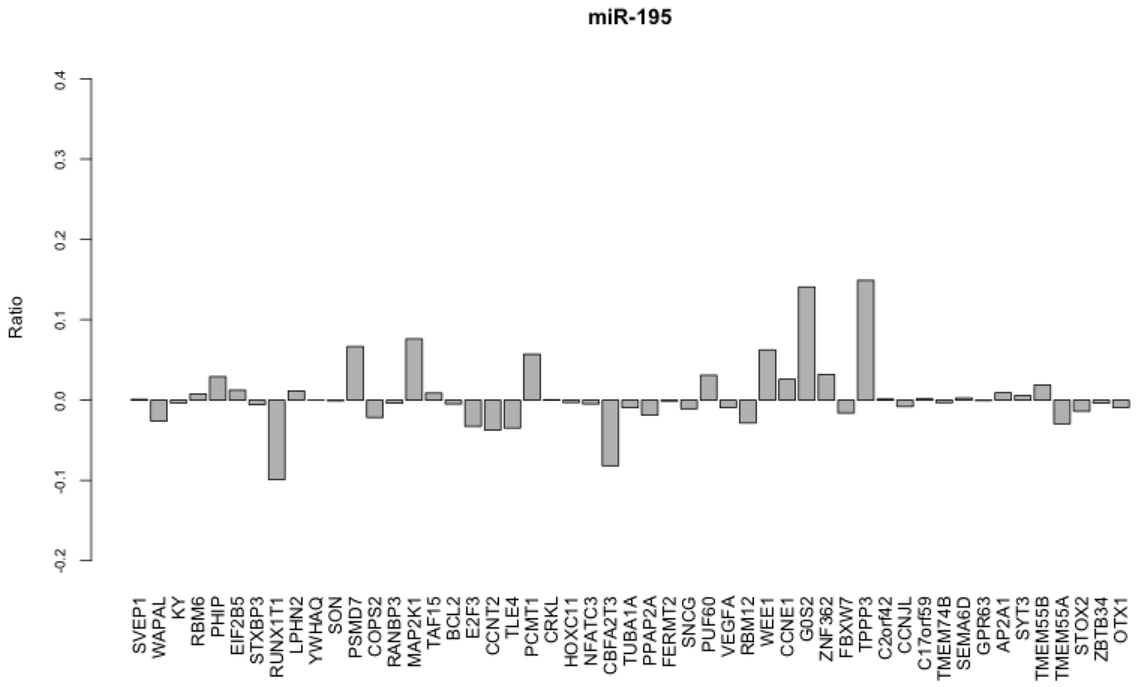

Supplementary Figure 9: Expression of the target genes of miR-195 in the *MLL*-rearrangement groups compared to that of the rest.

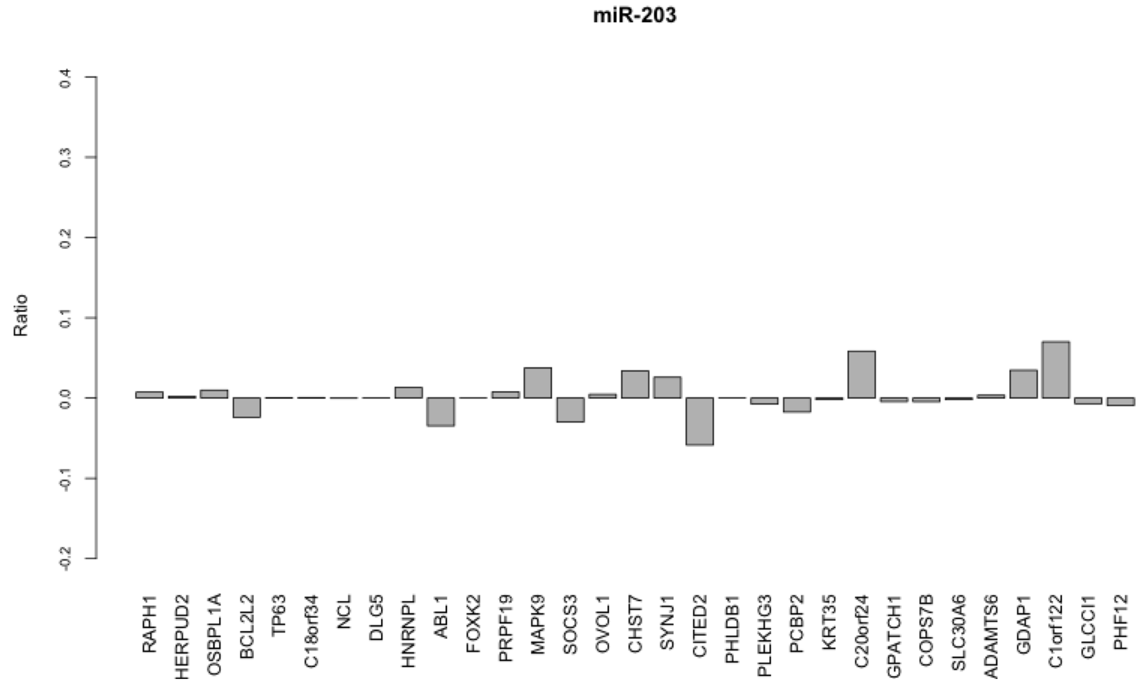

Supplementary Figure 10: Expression of the target genes of miR-203 in the *MLL*-rearrangement groups compared to that of the rest.

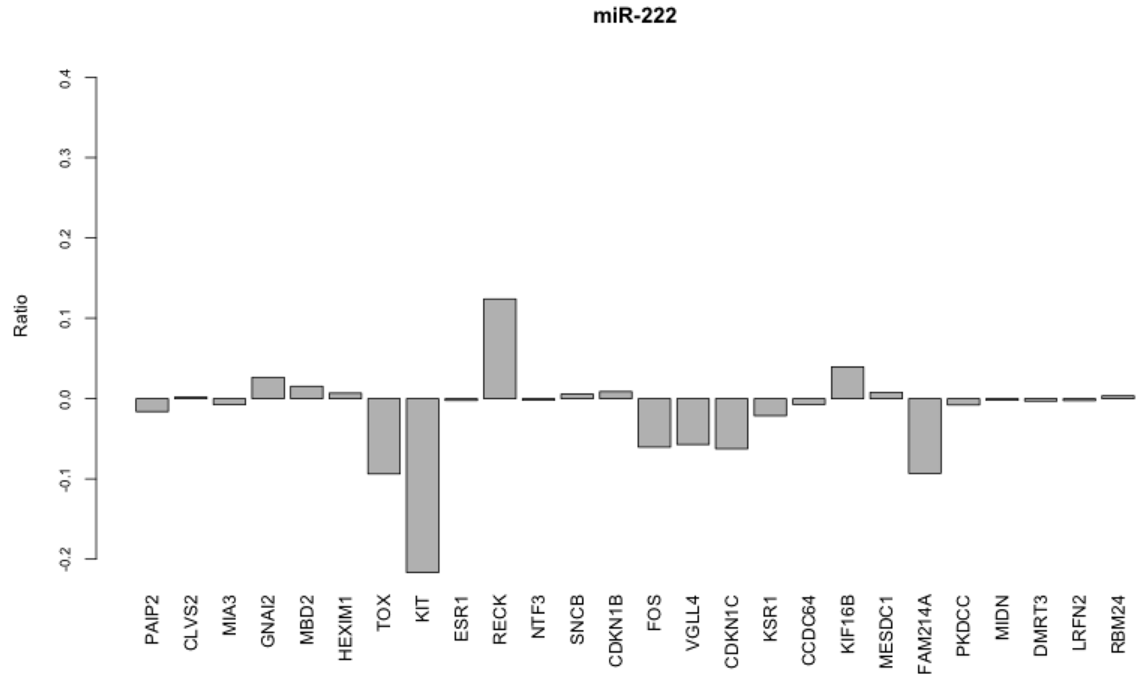

Supplementary Figure 11: Expression of the target genes of miR-222 in the *MLL*-rearrangement groups compared to that of the rest.

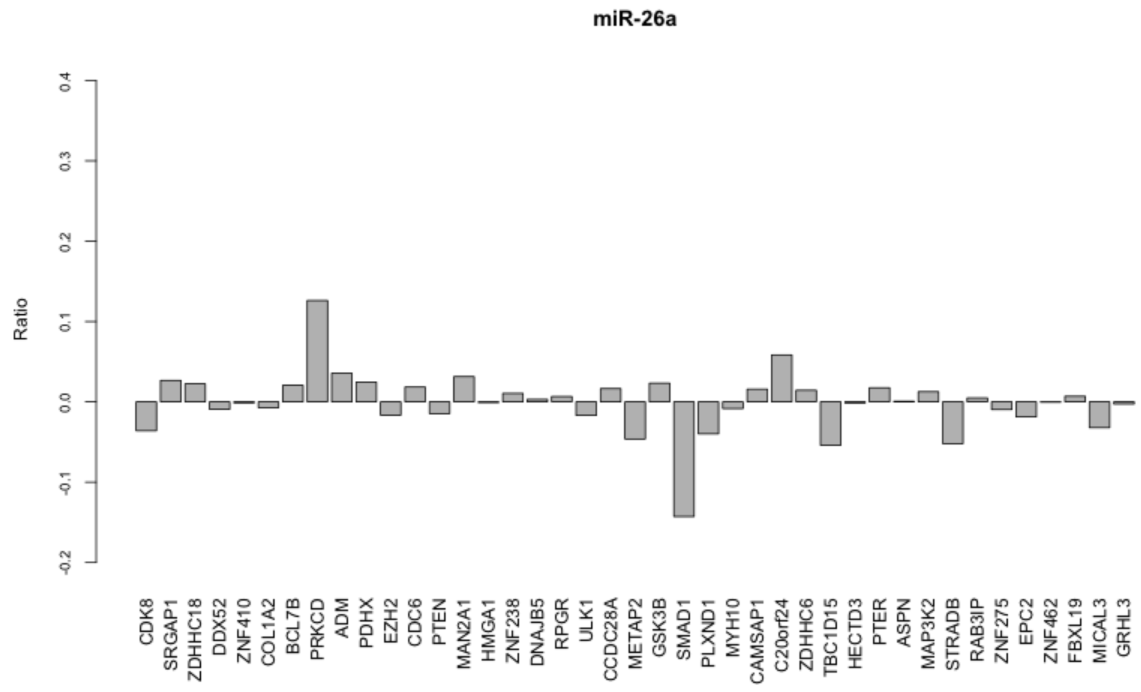

Supplementary Figure 12: Expression of the target genes of miR-26a in the *MLL*-rearrangement groups compared to that of the rest.

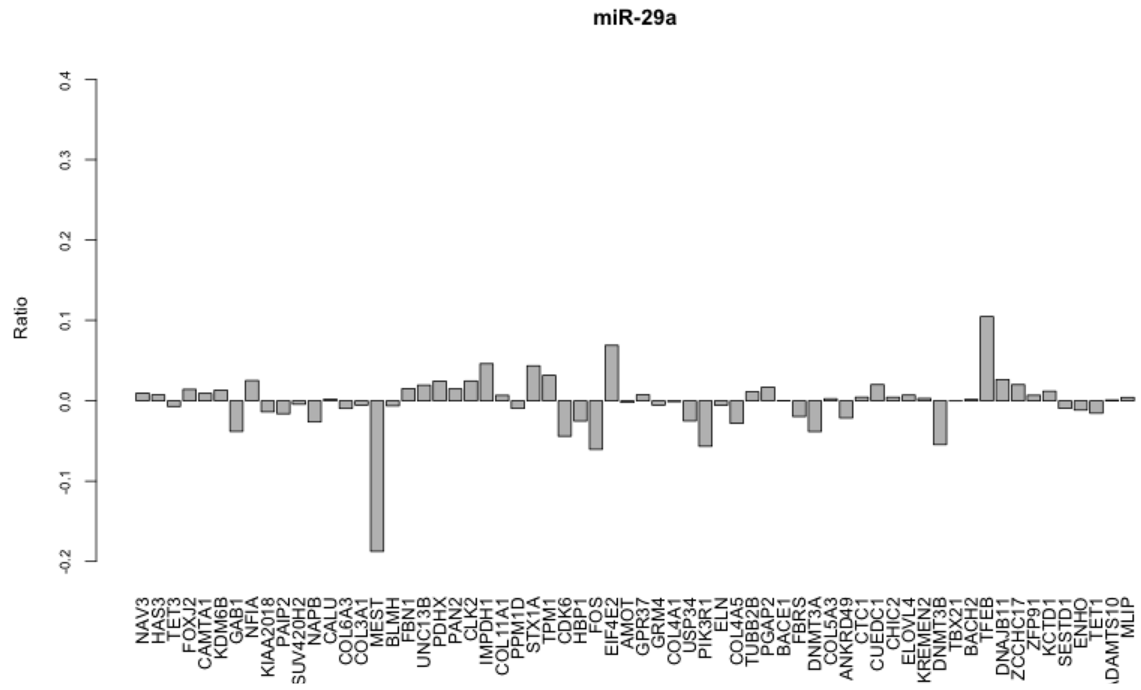

Supplementary Figure 13: Expression of the target genes of miR-29a in the *MLL*-rearrangement groups compared to that of the rest.

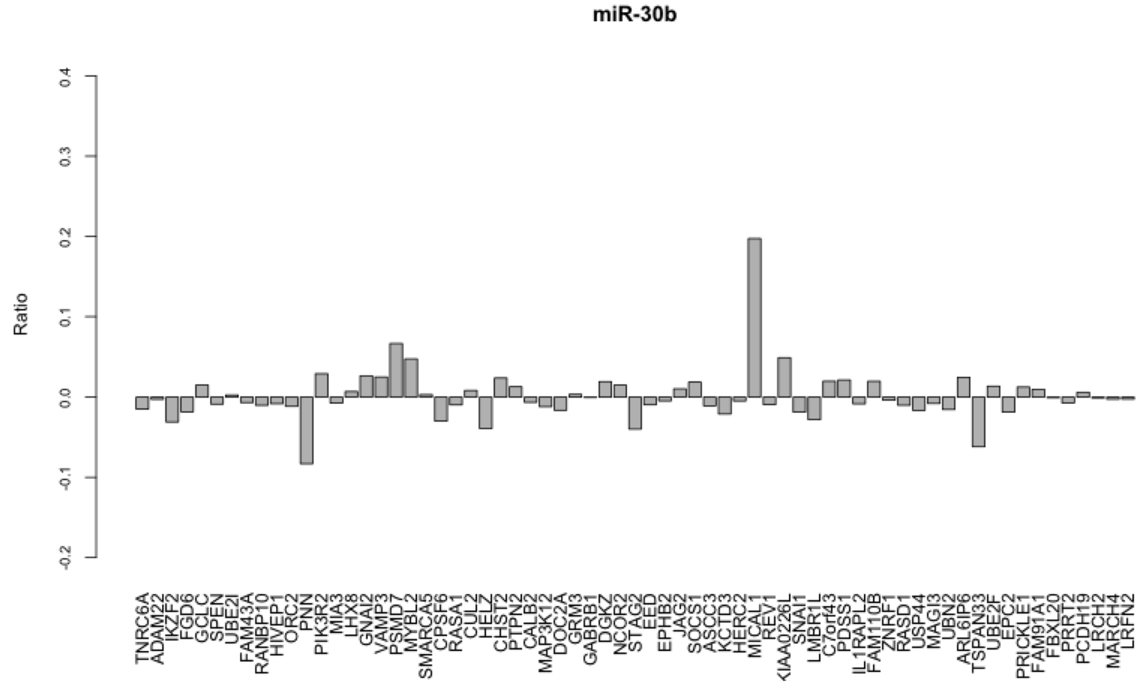

Supplementary Figure 14: Expression of the target genes of miR-30b in the *MLL*-rearrangement groups compared to that of the rest.

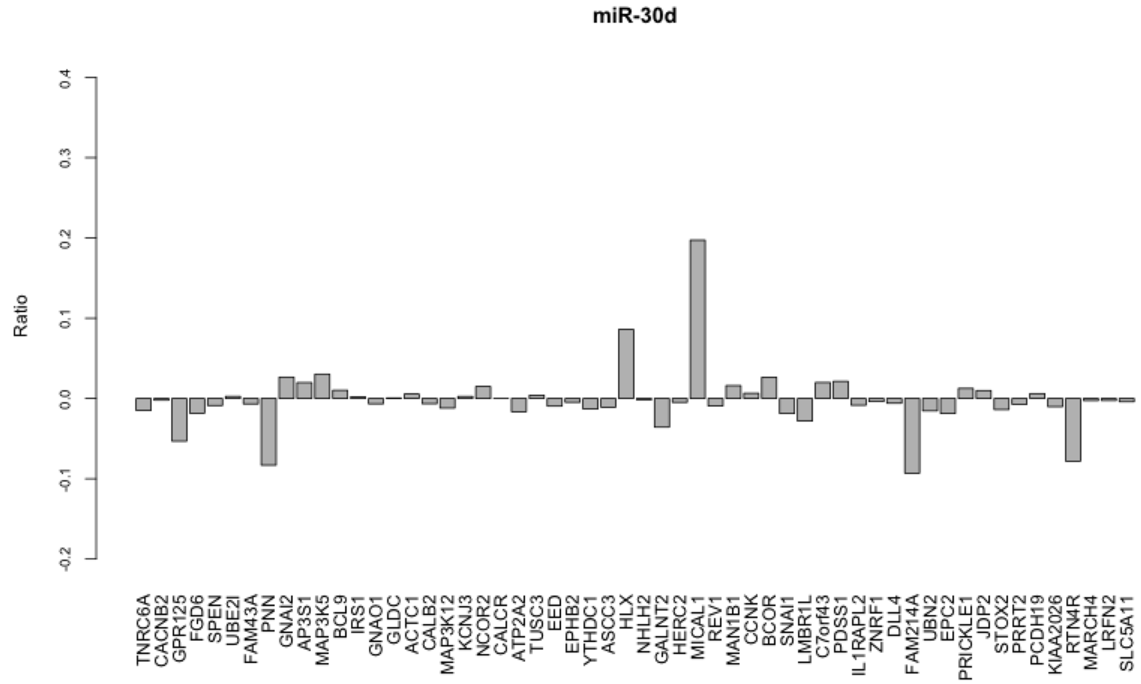

Supplementary Figure 15: Expression of the target genes of miR-30d in the *MLL*-rearrangement groups compared to that of the rest.

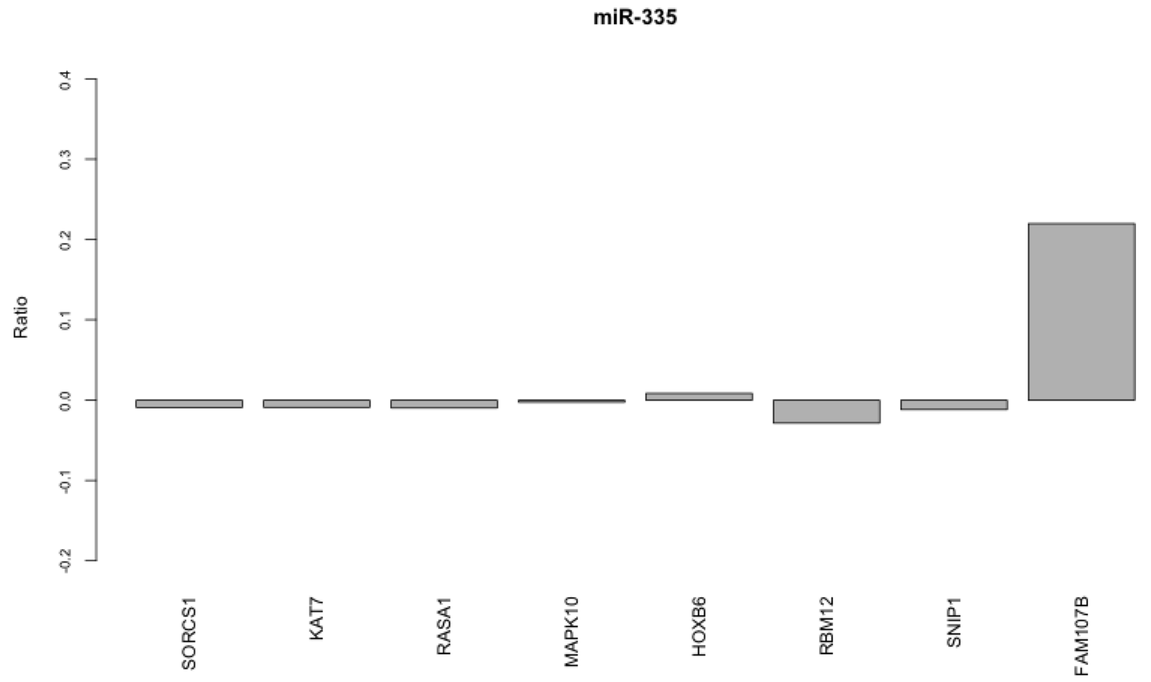

Supplementary Figure 16: Expression of the target genes of miR-335 in the *MLL*-rearrangement groups compared to that of the rest.

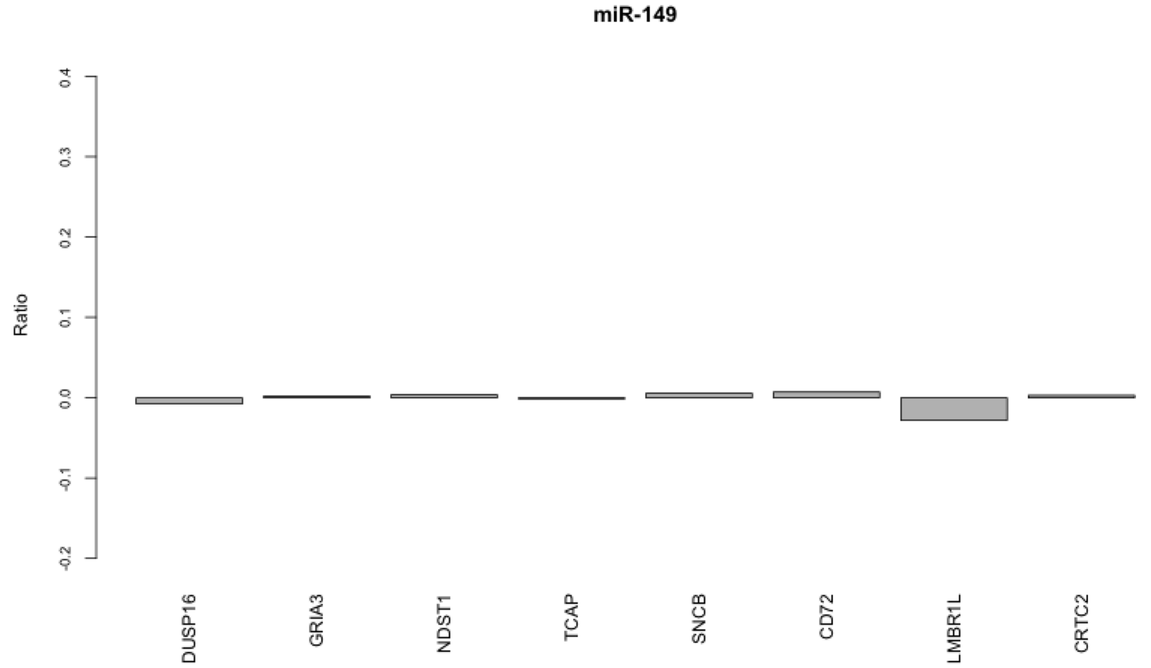

Supplementary Figure 17: Expression of the target genes of miR-149 in the *MLL*-rearrangement groups compared to that of the rest.

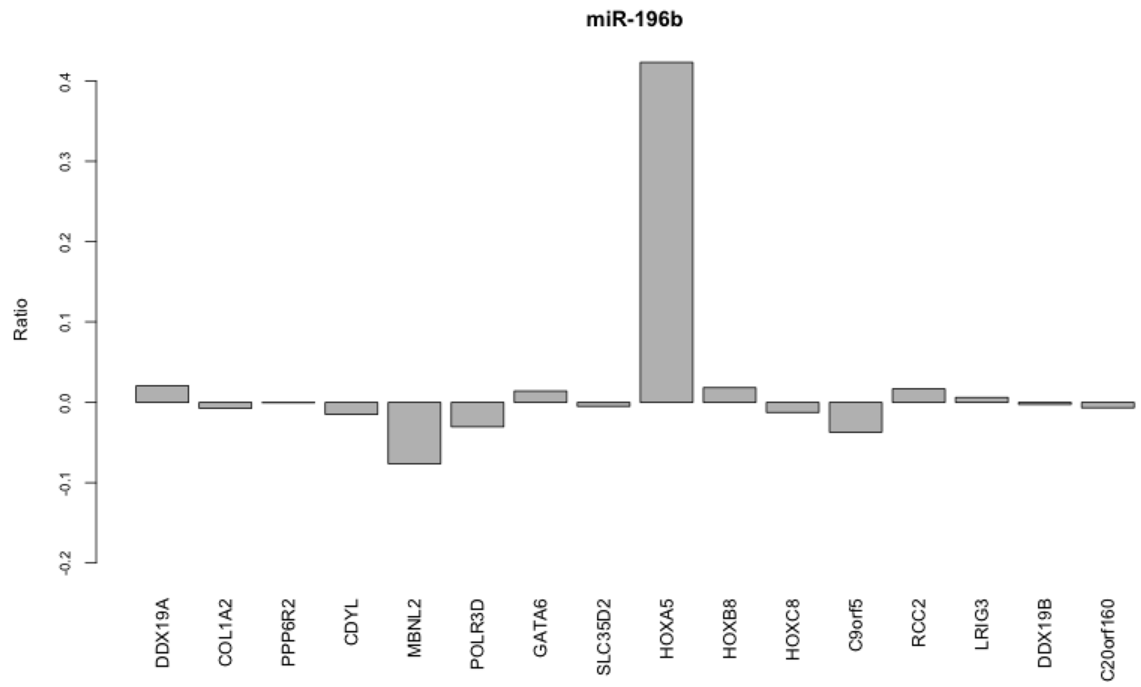

Supplementary Figure 18: Expression of the target genes of miR-196b in the *MLL*-rearrangement groups compared to that of the rest.

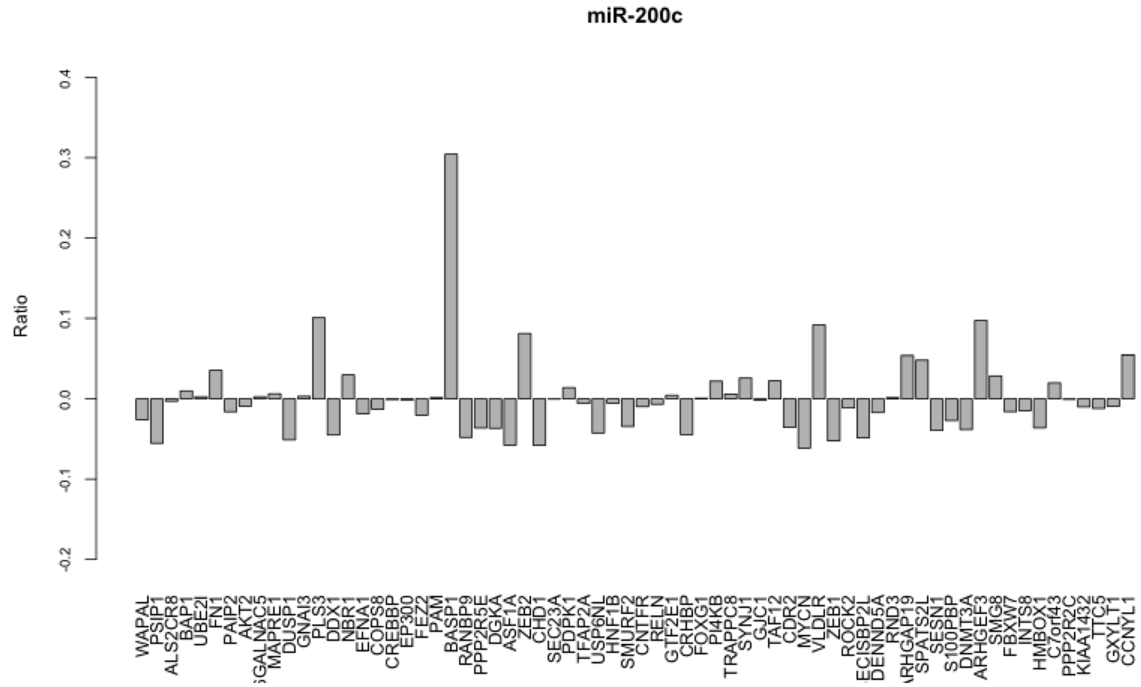

Supplementary Figure 19: Expression of the target genes of miR-200c in the *MLL*-rearrangement groups compared to that of the rest.

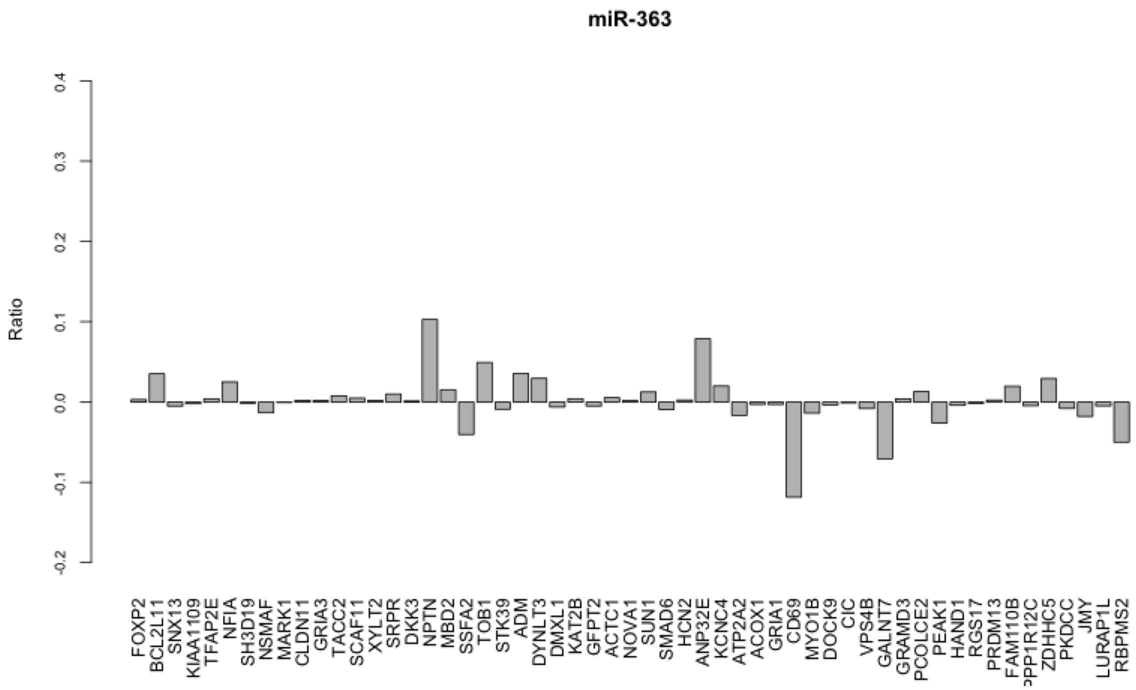

Supplementary Figure 20: Expression of the target genes of miR-363 in the *MLL*-rearrangement groups compared to that of the rest.

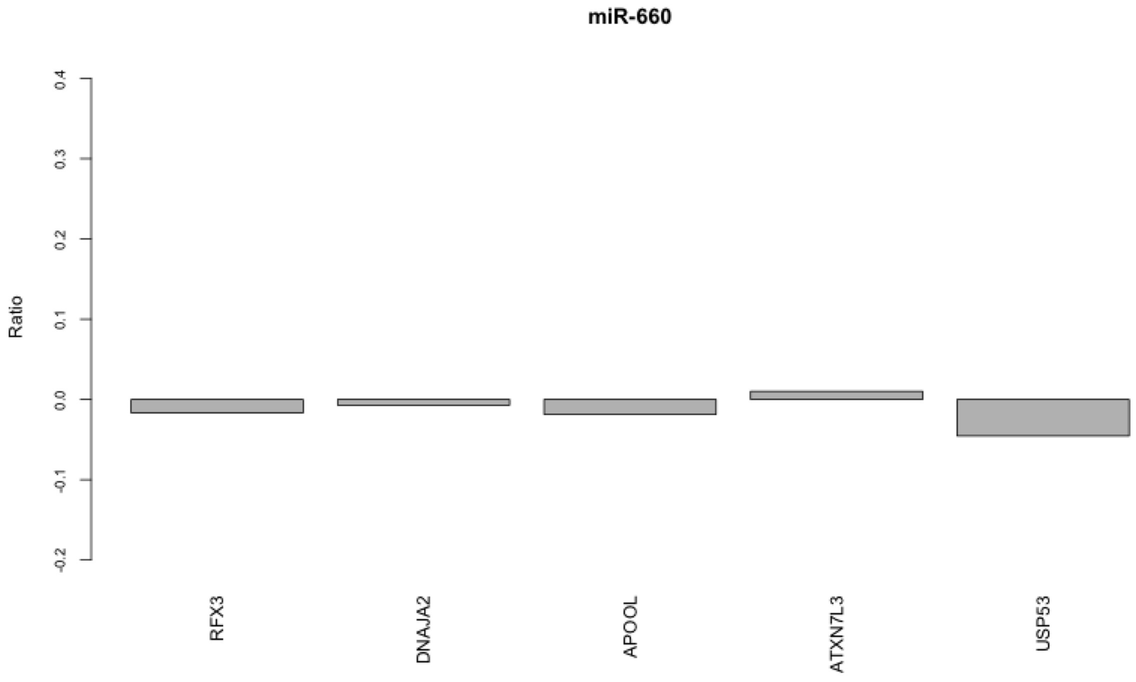

Supplementary Figure 21: Expression of the target genes of miR-660 in the *MLL*-rearrangement groups compared to that of the rest.

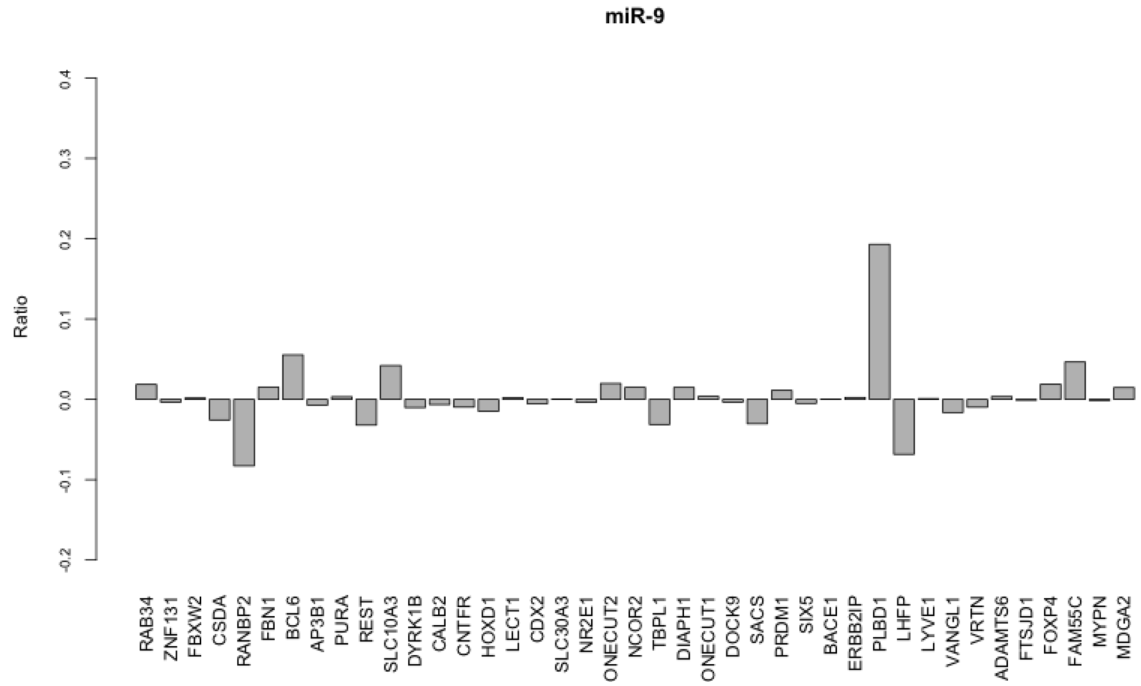

Supplementary Figure 22: Expression of the target genes of miR-9 in the *MLL*-rearrangement groups compared to that of the rest.

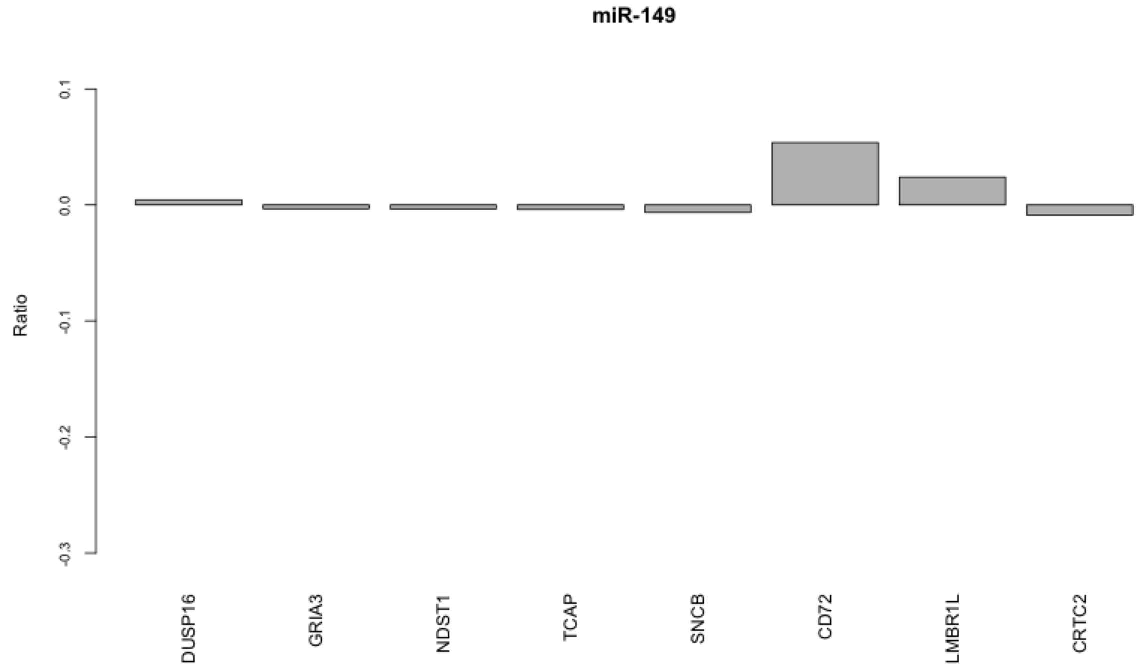

Supplementary Figure 23: Expression of the target genes of miR-149 in the t(8;21) groups compared to that of the rest.

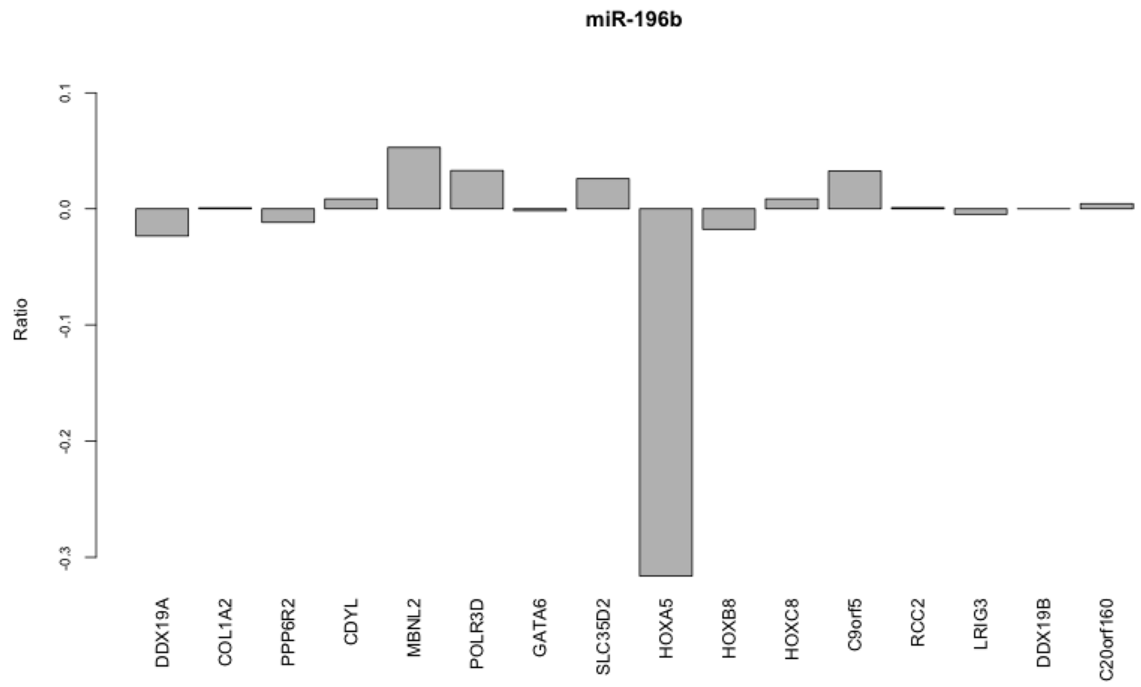

Supplementary Figure 24: Expression of the target genes of miR-196b in the t(8;21) groups compared to that of the rest.

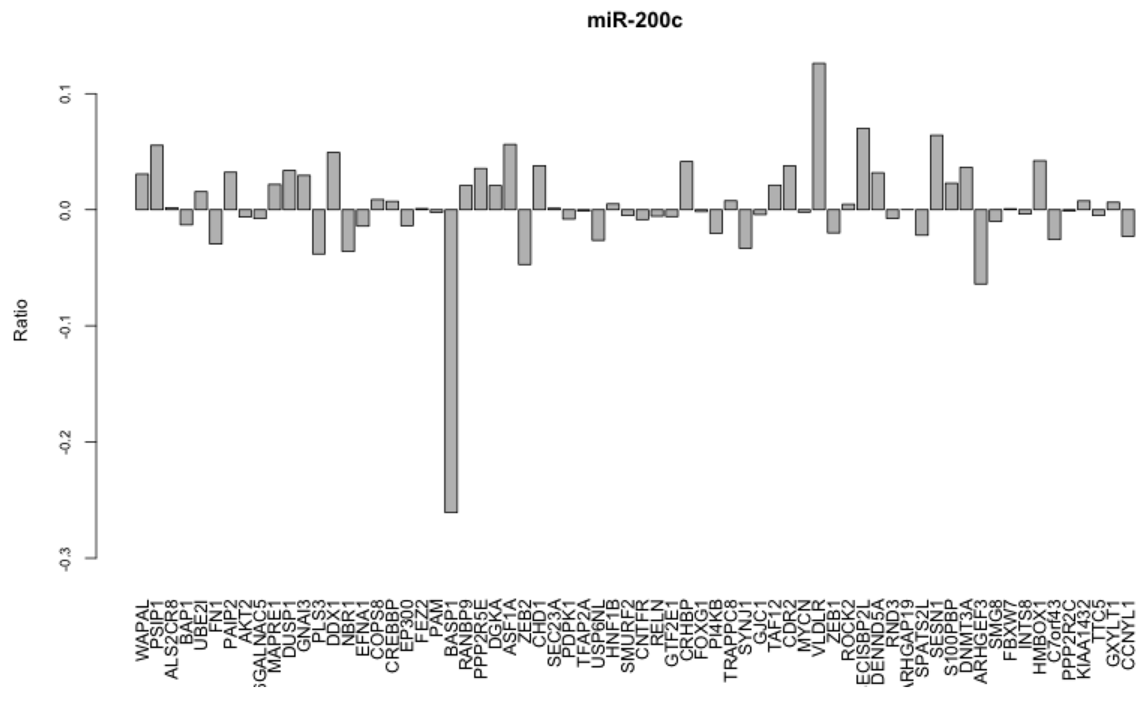

Supplementary Figure 25: Expression of the target genes of miR-200c in the t(8;21) groups compared to that of the rest.

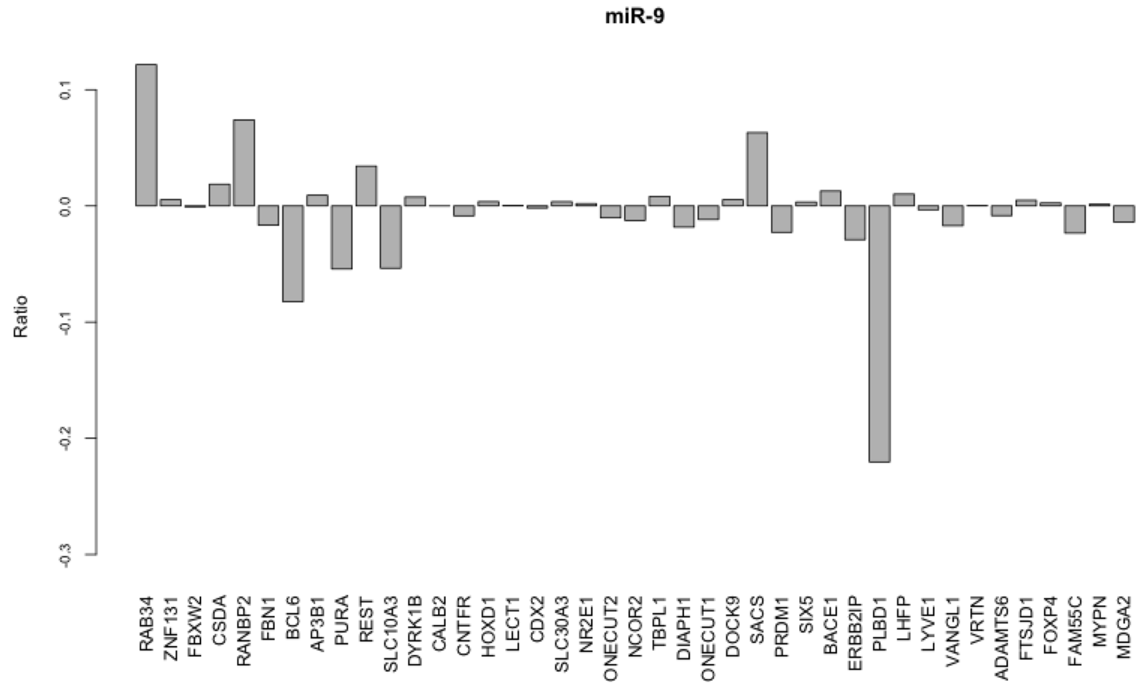

Supplementary Figure 26: Expression of the target genes of miR-9 in the t(8;21) groups compared to that of the rest.

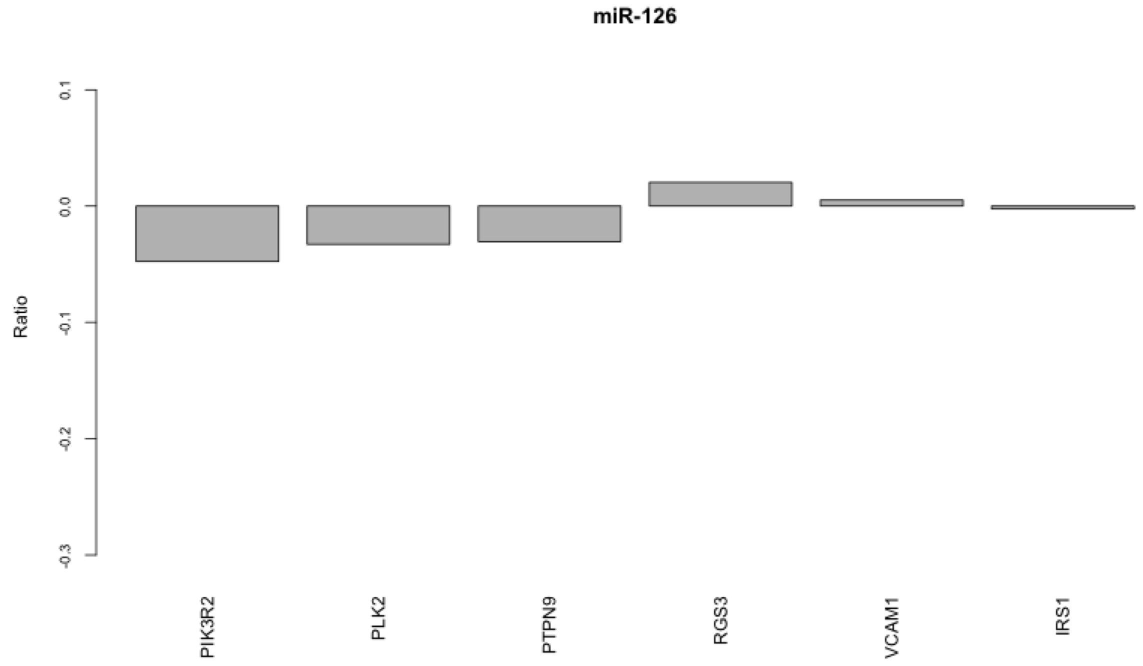

Supplementary Figure 27: Expression of the target genes of miR-126 in the t(8;21) groups compared to that of the rest.

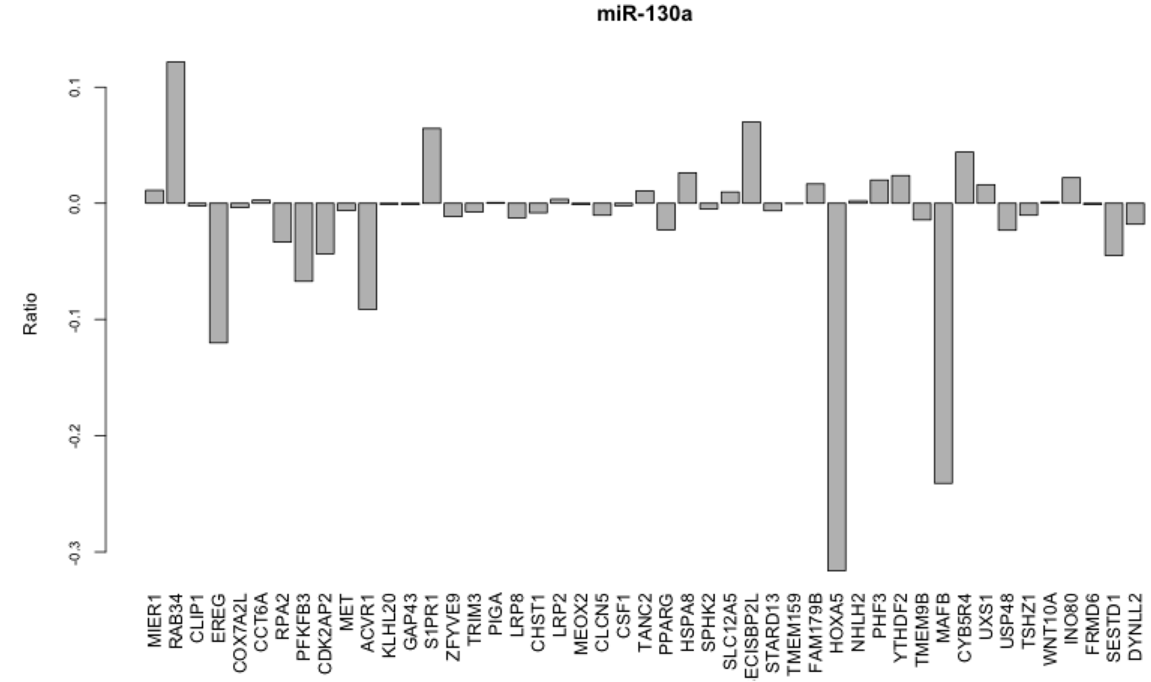

Supplementary Figure 28: Expression of the target genes of miR-130a in the t(8;21) groups compared to that of the rest.

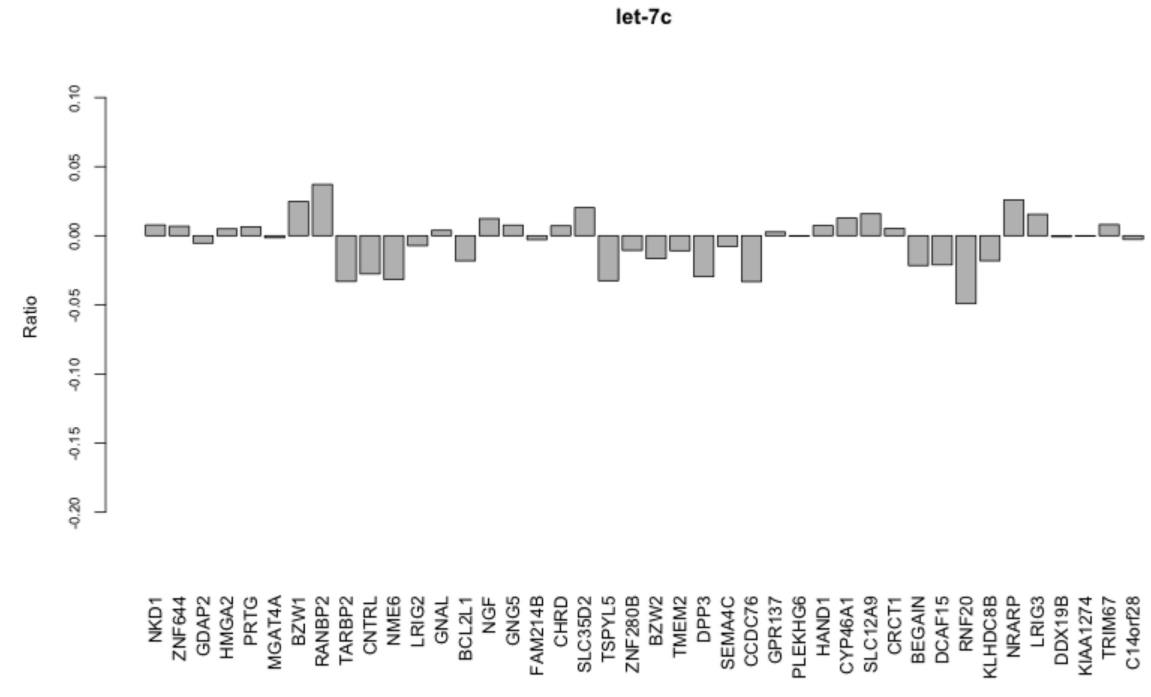

Supplementary Figure 29: Expression of the target genes of let-7c in the inv(16) groups compared to that of the rest.

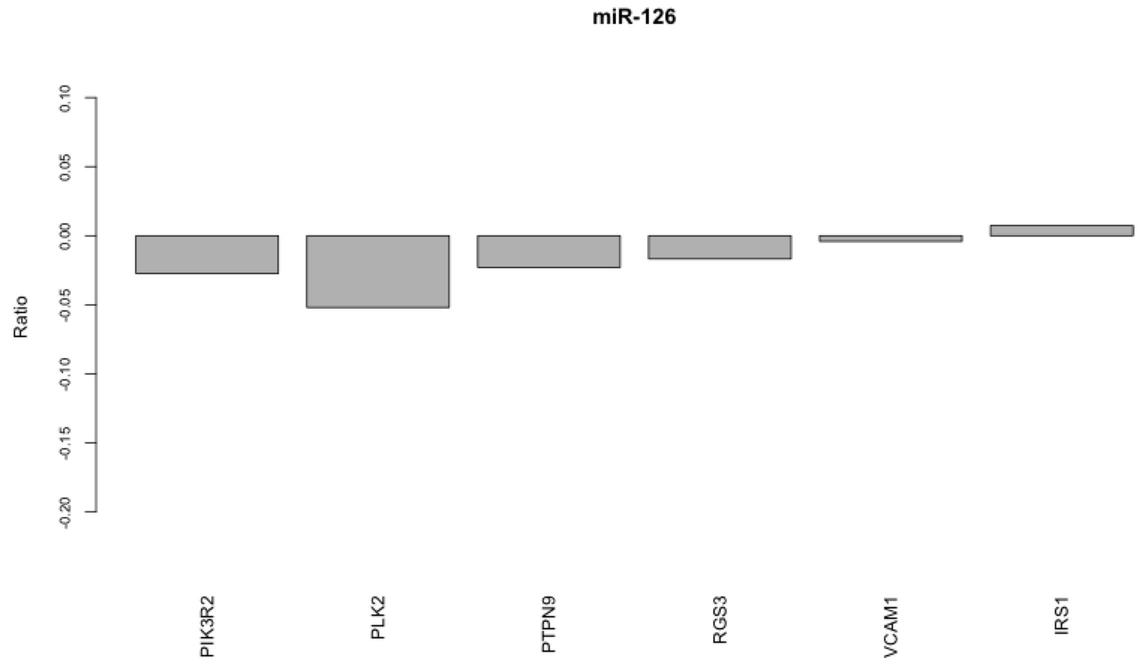

Supplementary Figure 30: Expression of the target genes of miR-126 in the inv(16) groups compared to that of the rest.

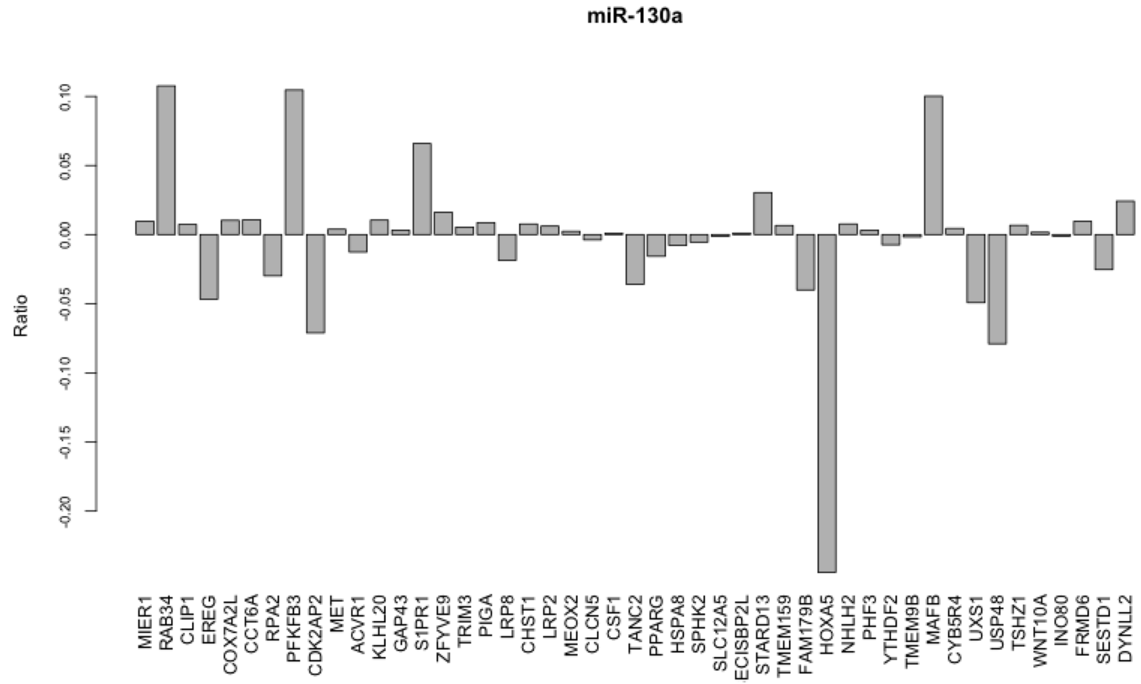

Supplementary Figure 31: Expression of the target genes of miR-130a in the inv(16) groups compared to that of the rest.

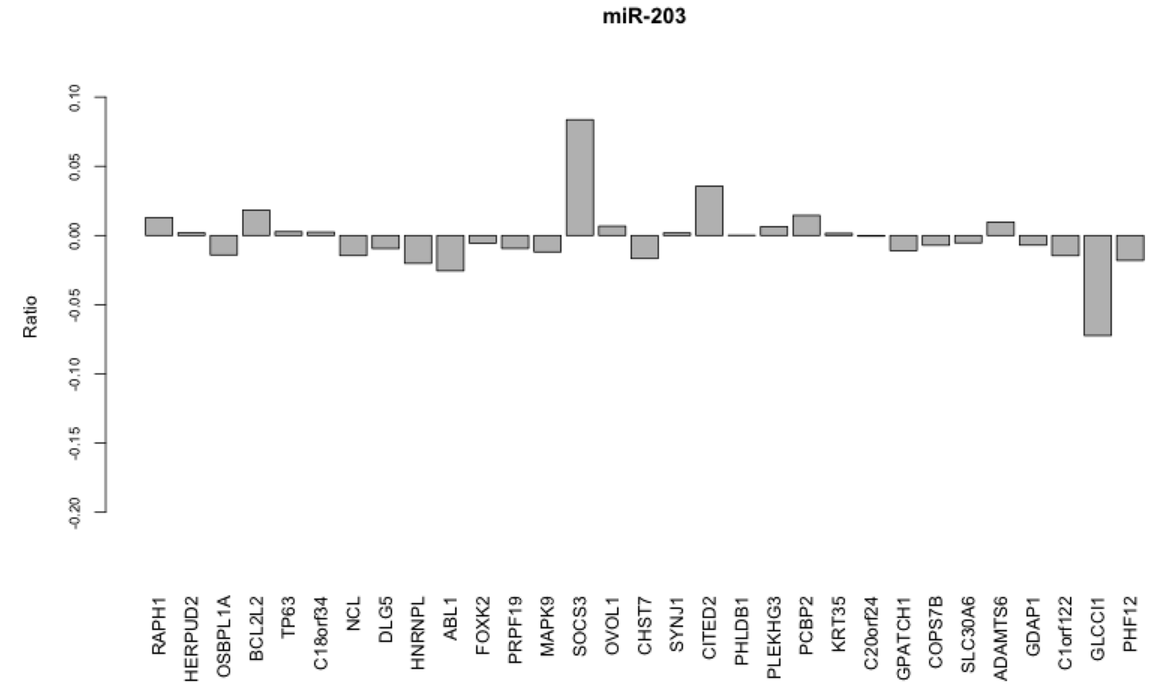

Supplementary Figure 32: Expression of the target genes of miR-203 in the inv(16) groups compared to that of the rest.

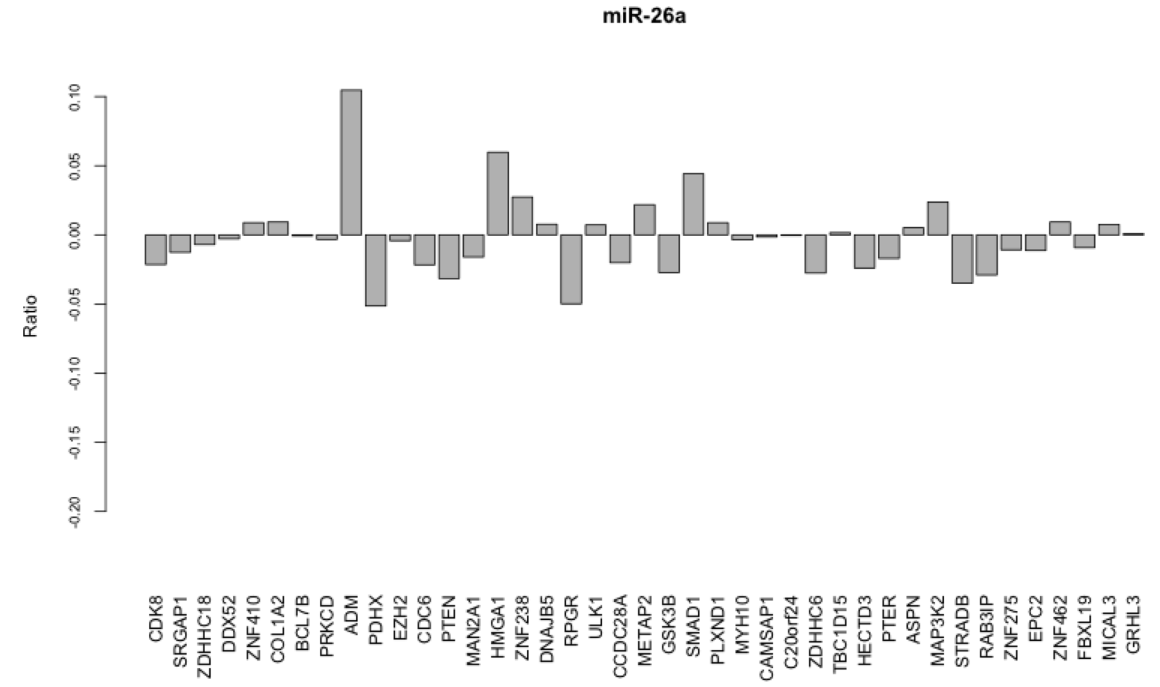

Supplementary Figure 33: Expression of the target genes of miR-26a in the inv(16) groups compared to that of the rest.

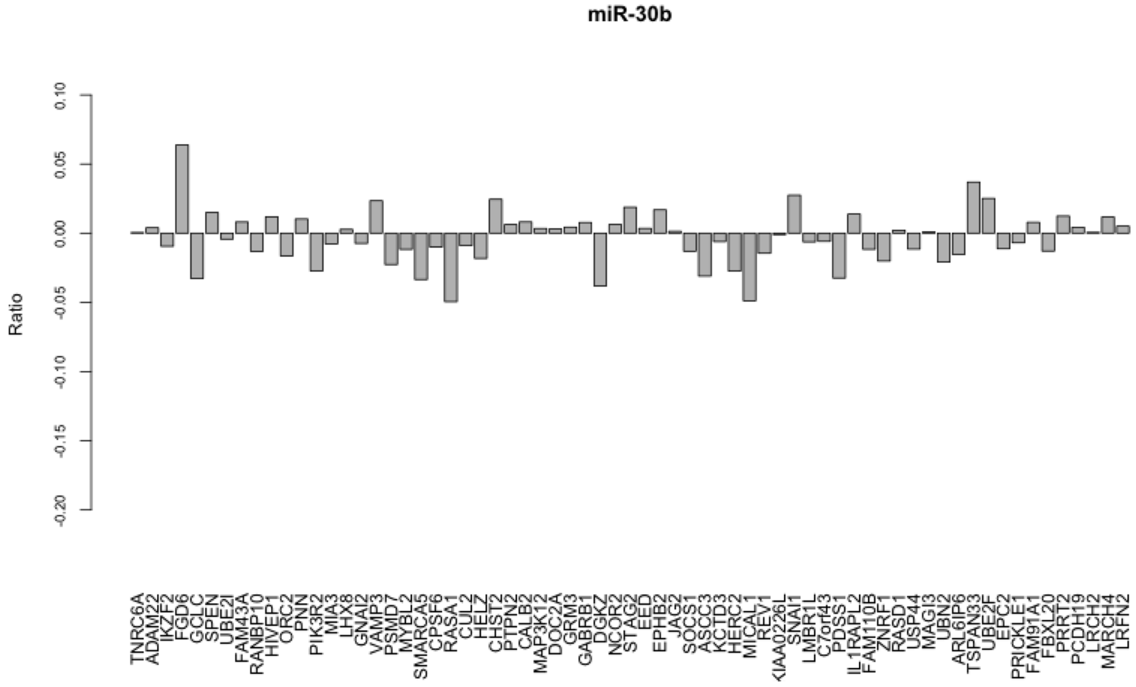

Supplementary Figure 34: Expression of the target genes of miR-30b in the inv(16) groups compared to that of the rest.

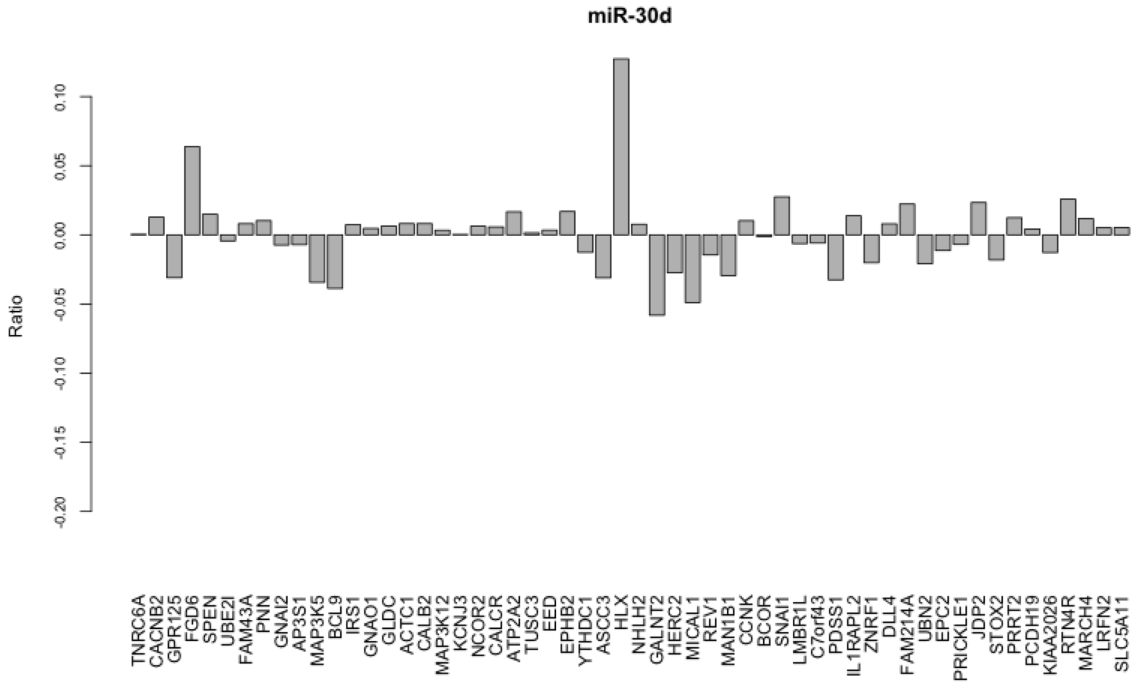

Supplementary Figure 35: Expression of the target genes of miR-30d in the inv(16) groups compared to that of the rest.

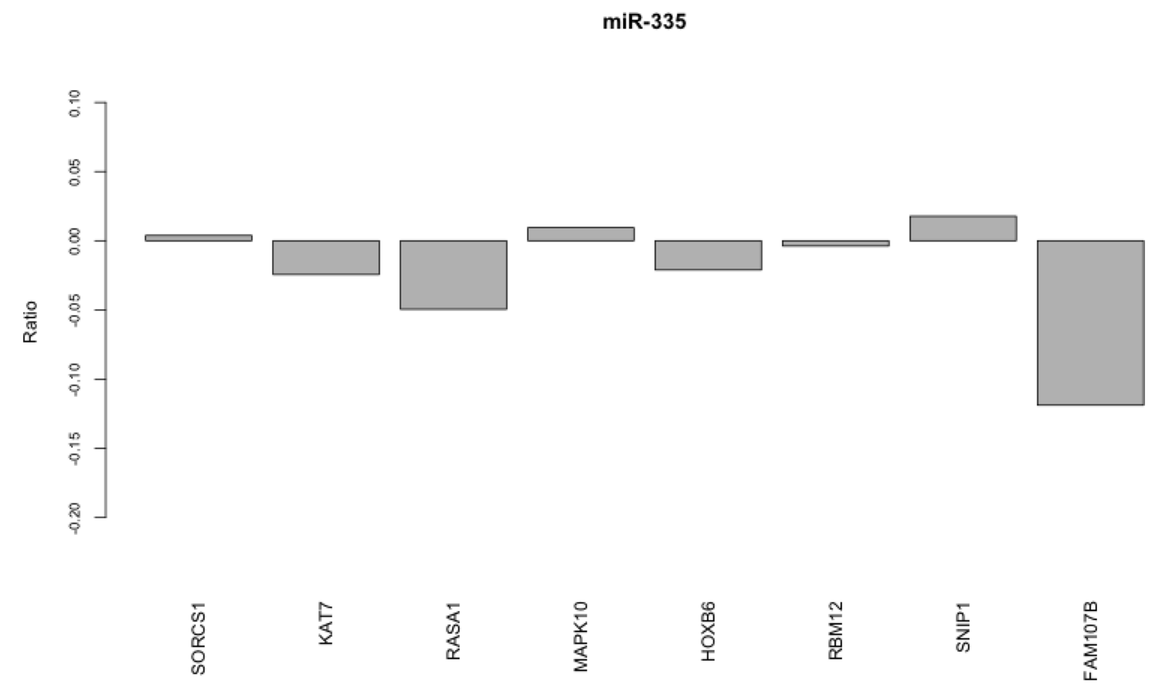

Supplementary Figure 36: Expression of the target genes of miR-335 in the inv(16) groups compared to that of the rest.

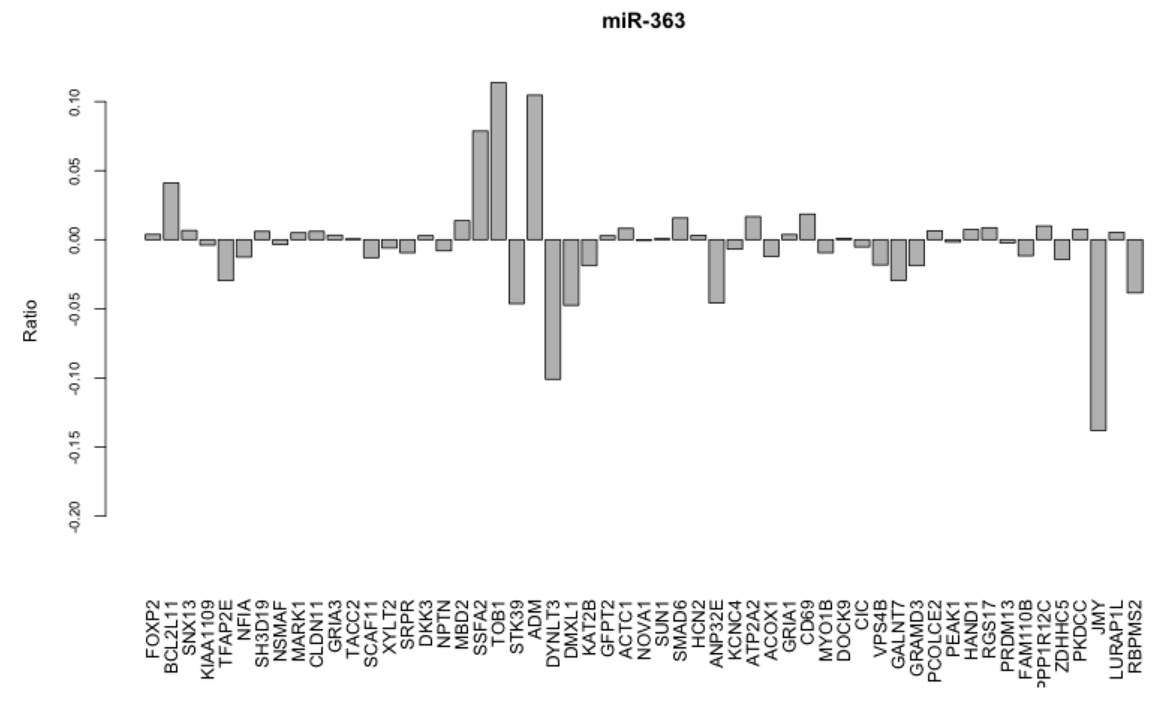

Supplementary Figure 37: Expression of the target genes of miR-363 in the inv(16) groups compared to that of the rest.

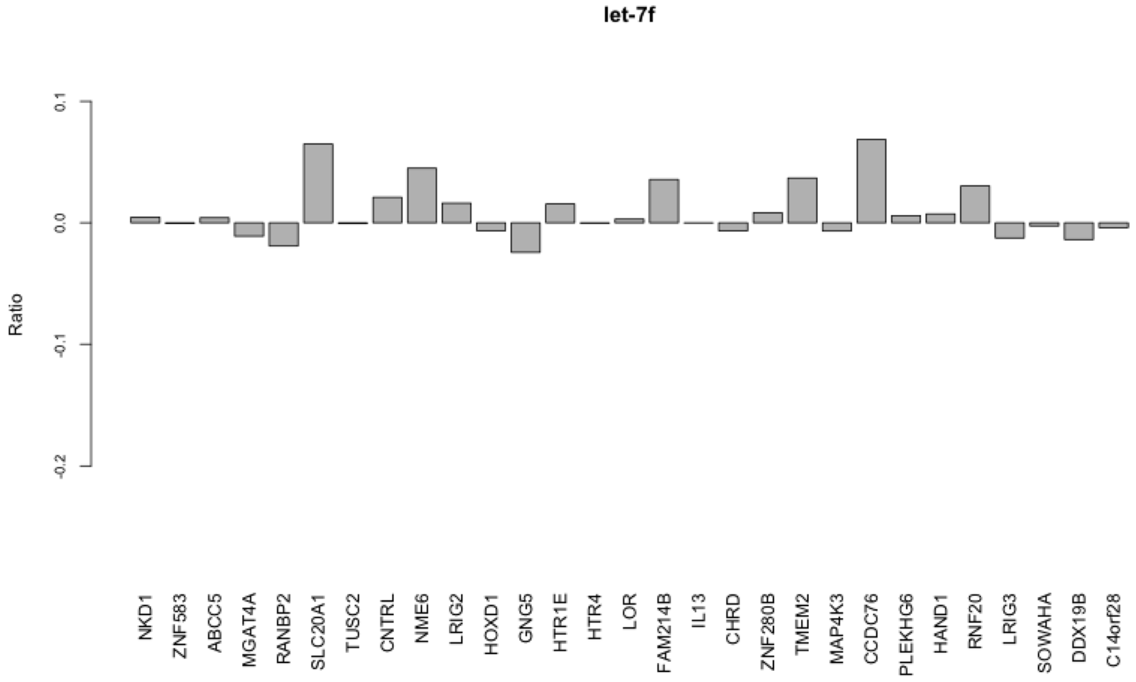

Supplementary Figure 38: Expression of the target genes of let-7c in the t(15;17) groups compared to that of the rest.

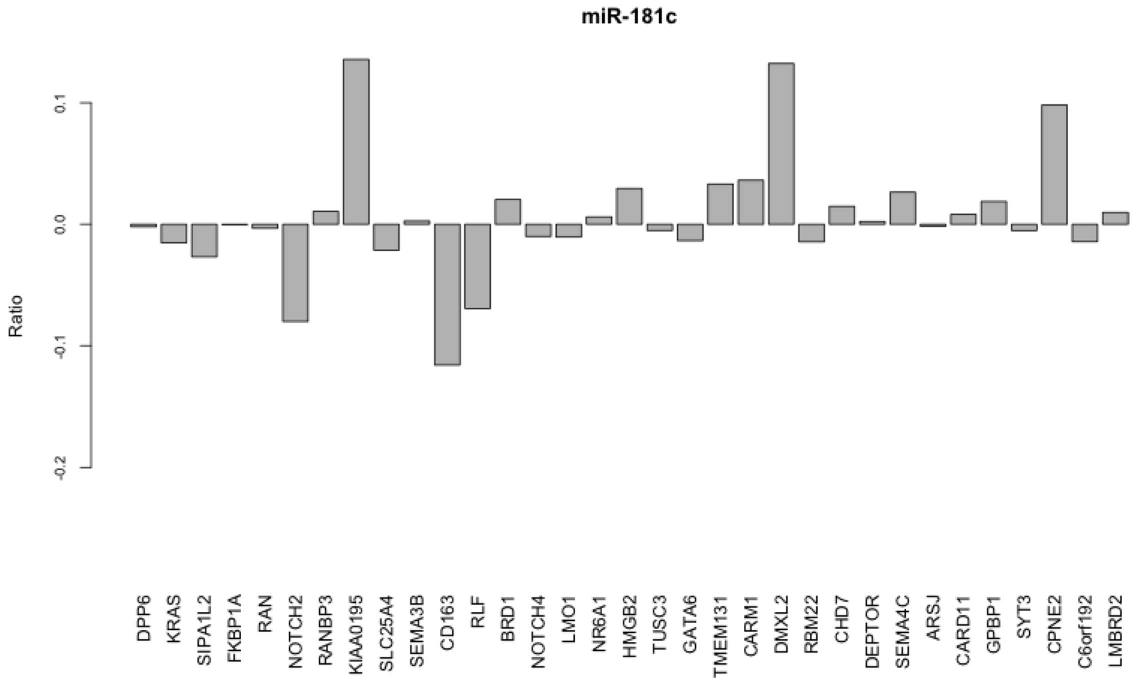

Supplementary Figure 39: Expression of the target genes of miR-181c in the t(15;17) groups compared to that of the rest.

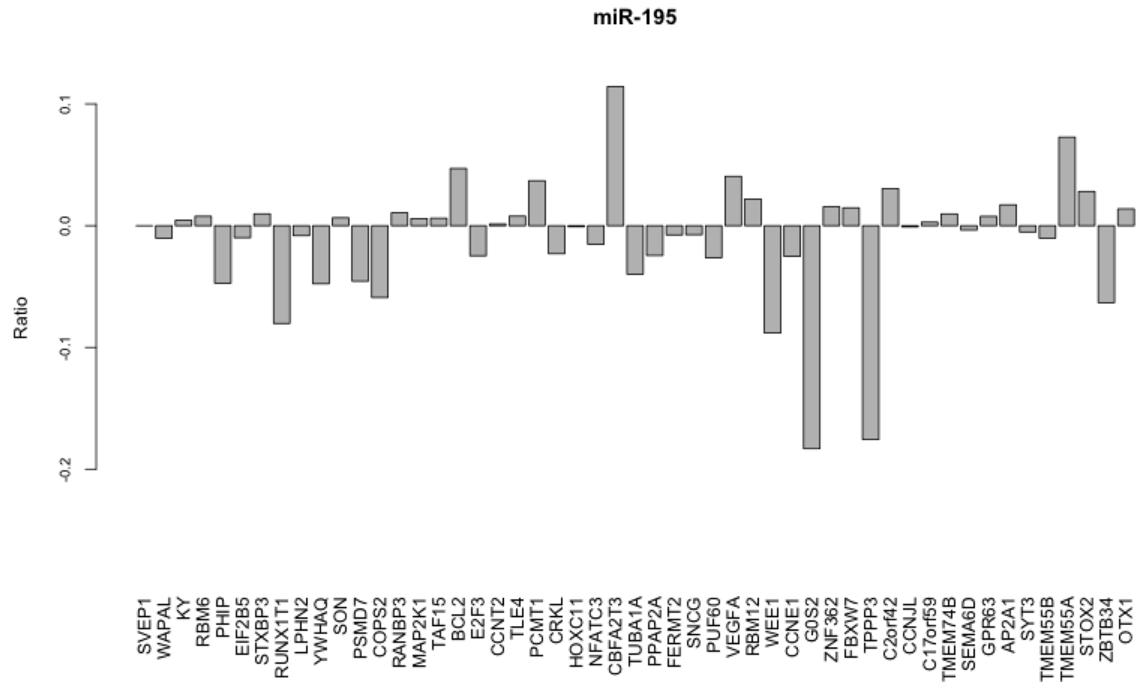

Supplementary Figure 40: Expression of the target genes of miR-195 in the t(15;17) groups compared to that of the rest.

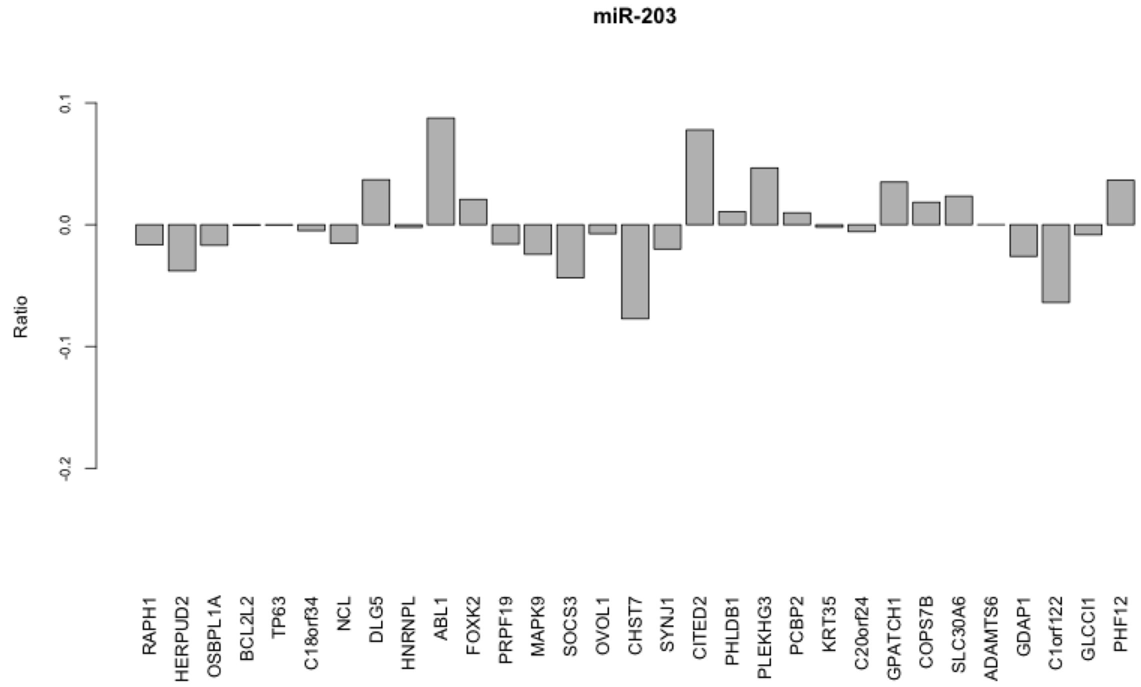

Supplementary Figure 41: Expression of the target genes of miR-203 in the t(15;17) groups compared to that of the rest.

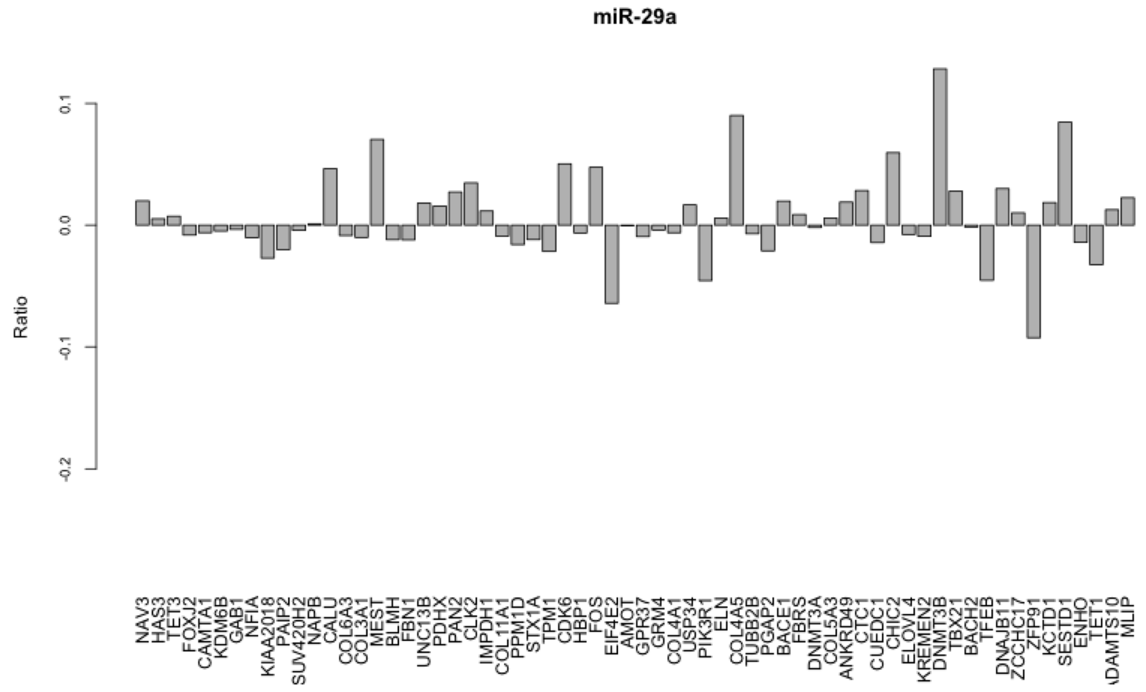

Supplementary Figure 42: Expression of the target genes of miR-29a in the t(15;17) groups compared to that of the rest.

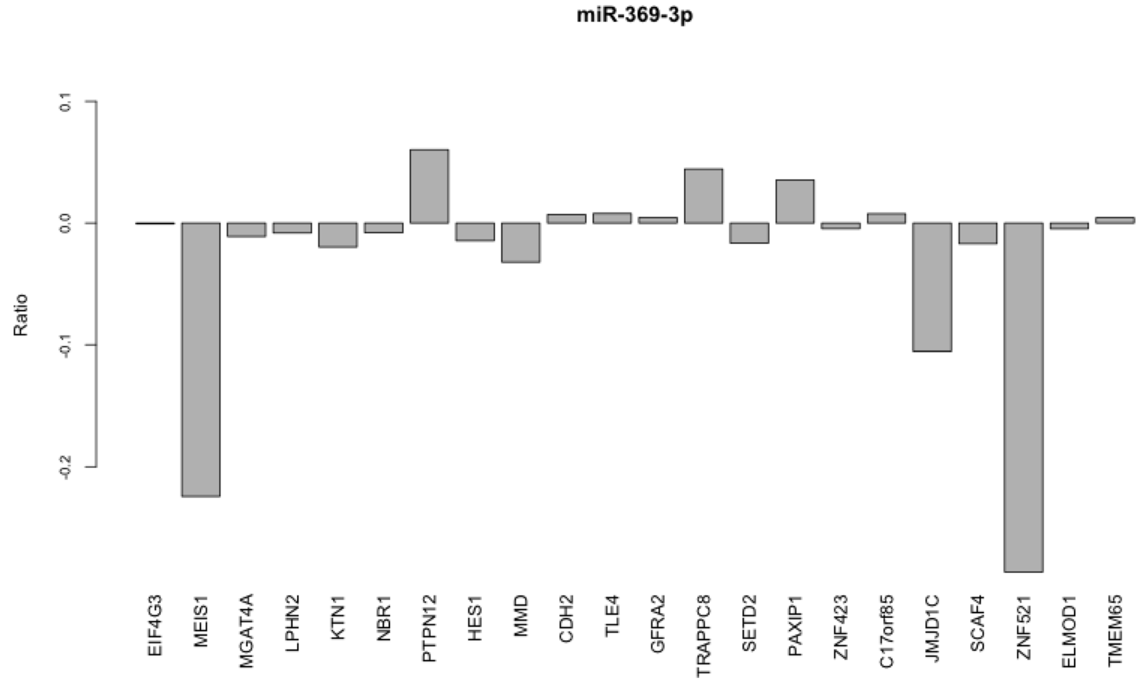

Supplementary Figure 43: Expression of the target genes of miR-369-3p in the t(15;17) groups compared to that of the rest.

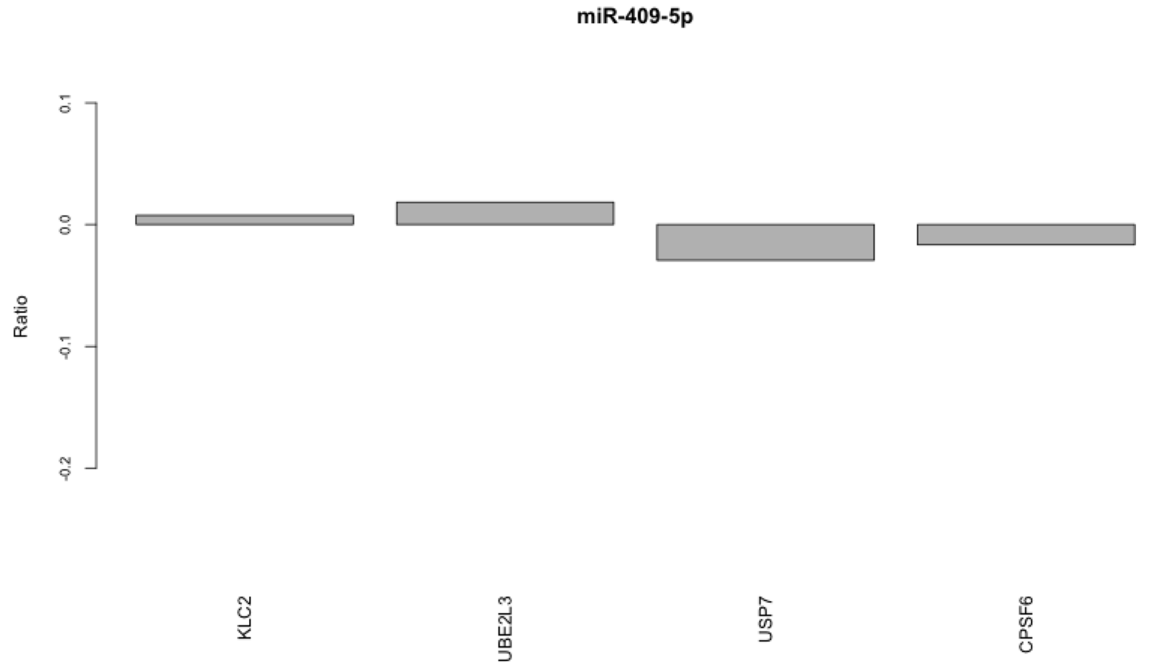

Supplementary Figure 44: Expression of the target genes of miR-409-5p in the t(15;17) groups compared to that of the rest.

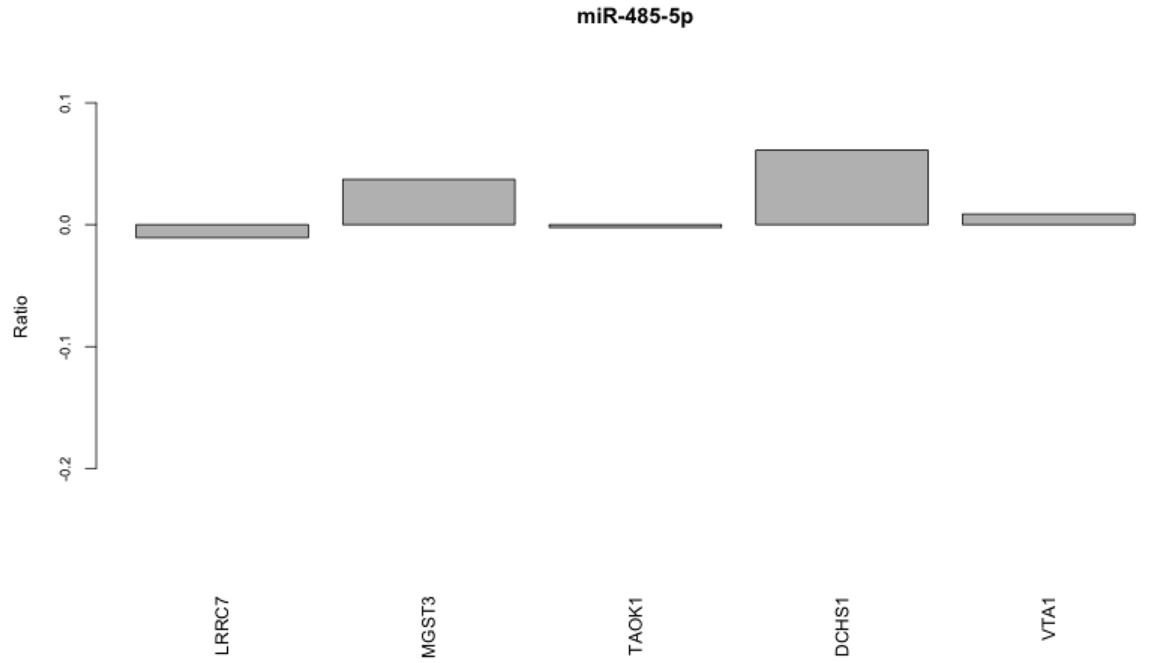

Supplementary Figure 45: Expression of the target genes of miR-485-5p in the t(15;17) groups compared to that of the rest.

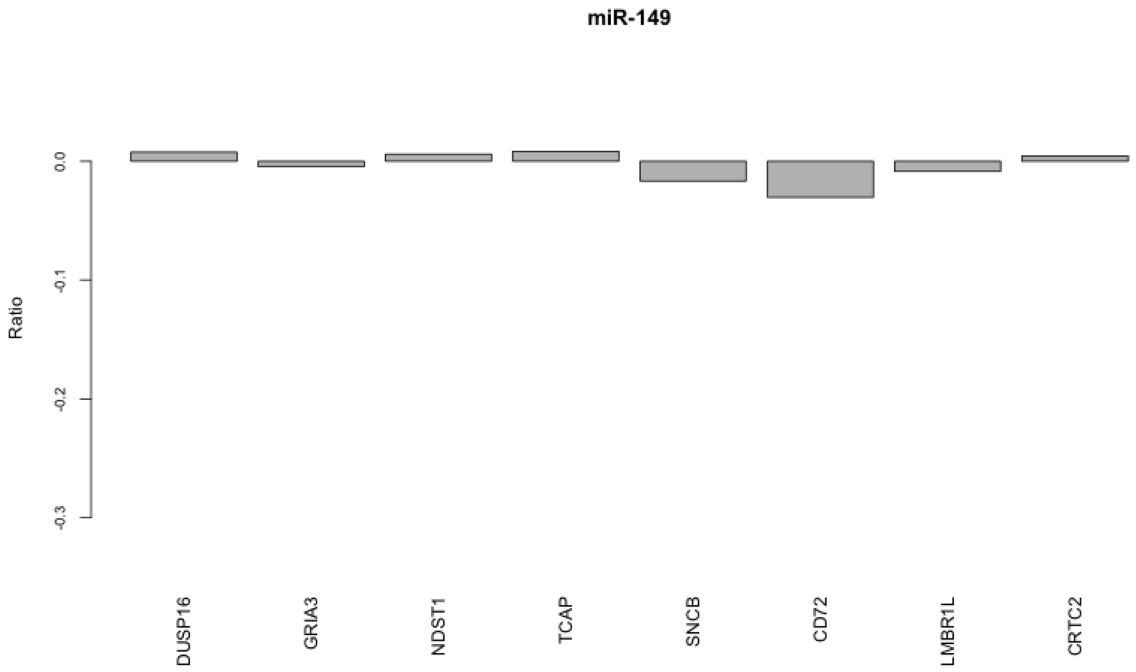

Supplementary Figure 46: Expression of the target genes of miR-149 in the CEPBA.dm groups compared to that of the rest.

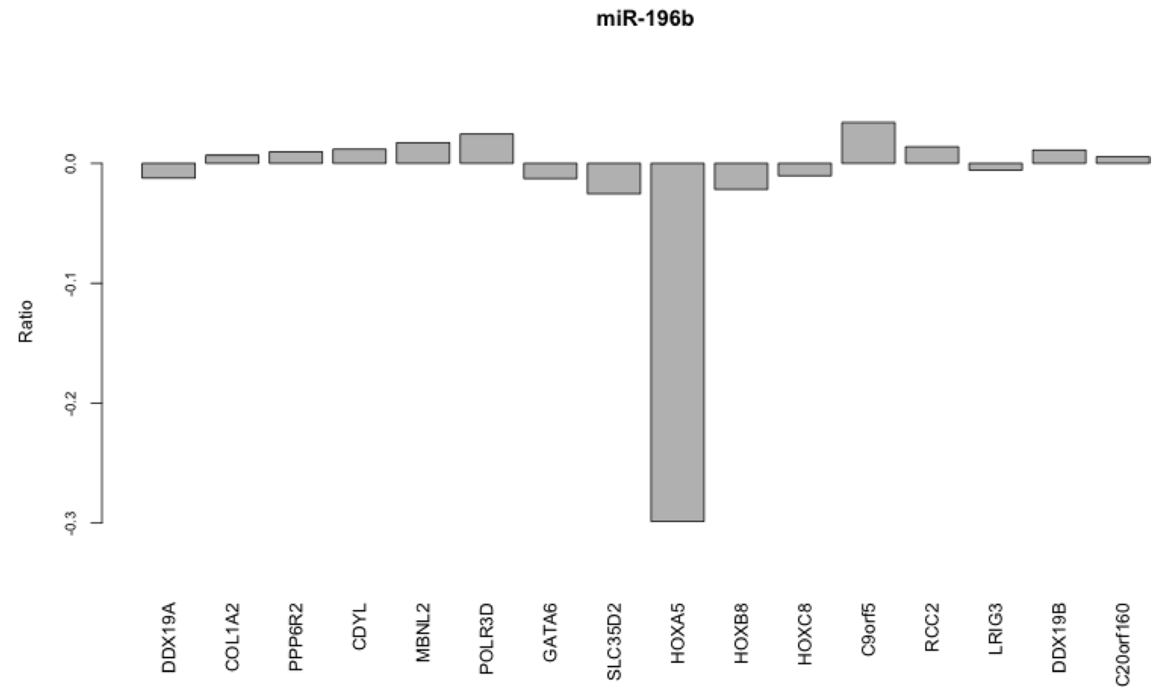

Supplementary Figure 47: Expression of the target genes of miR-196b in the CEPBA.dm groups compared to that of the rest.

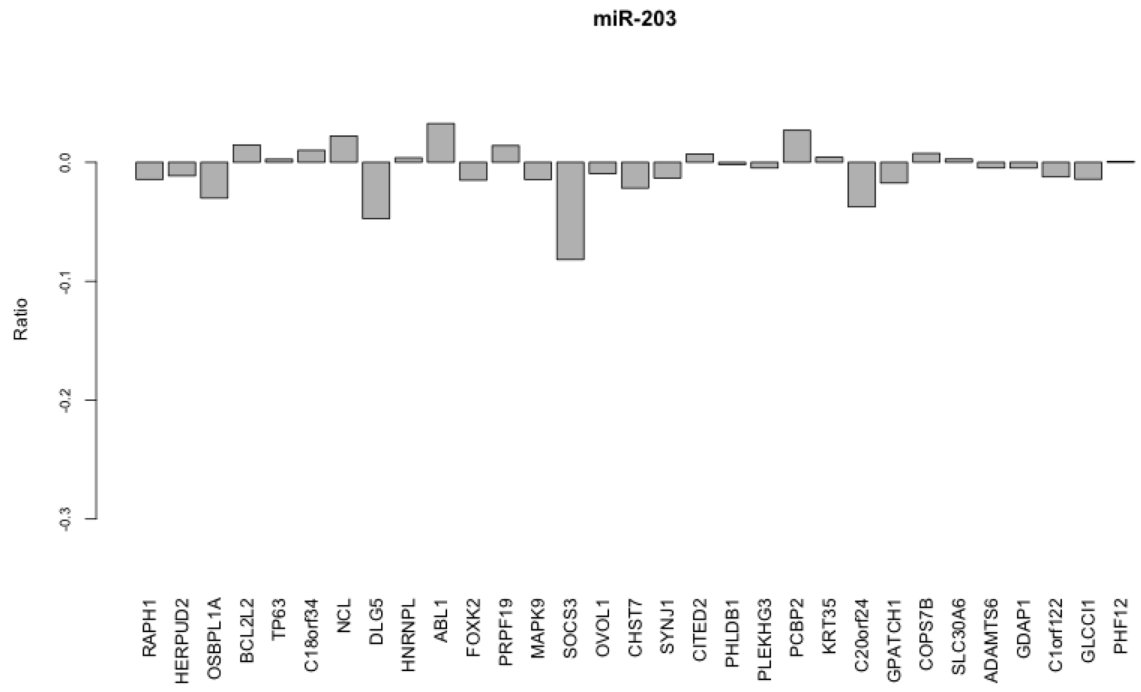

Supplementary Figure 48: Expression of the target genes of miR-203 in the CEPBA.dm groups compared to that of the rest.

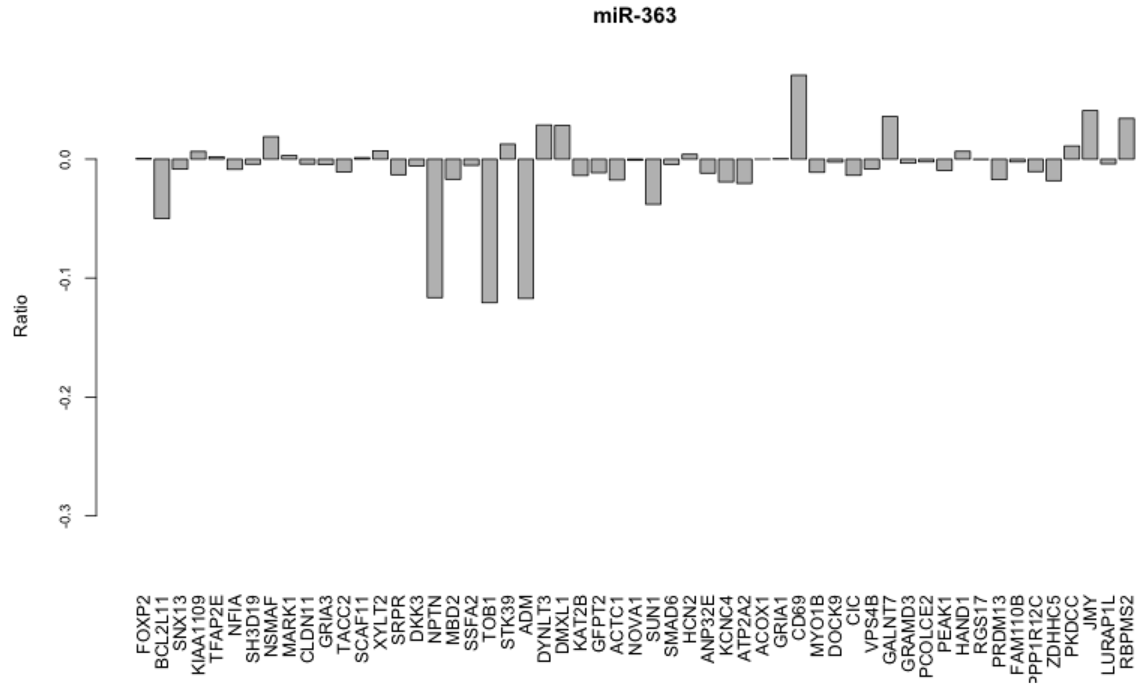

Supplementary Figure 49: Expression of the target genes of miR-363 in the CEPBA.dm groups compared to that of the rest.

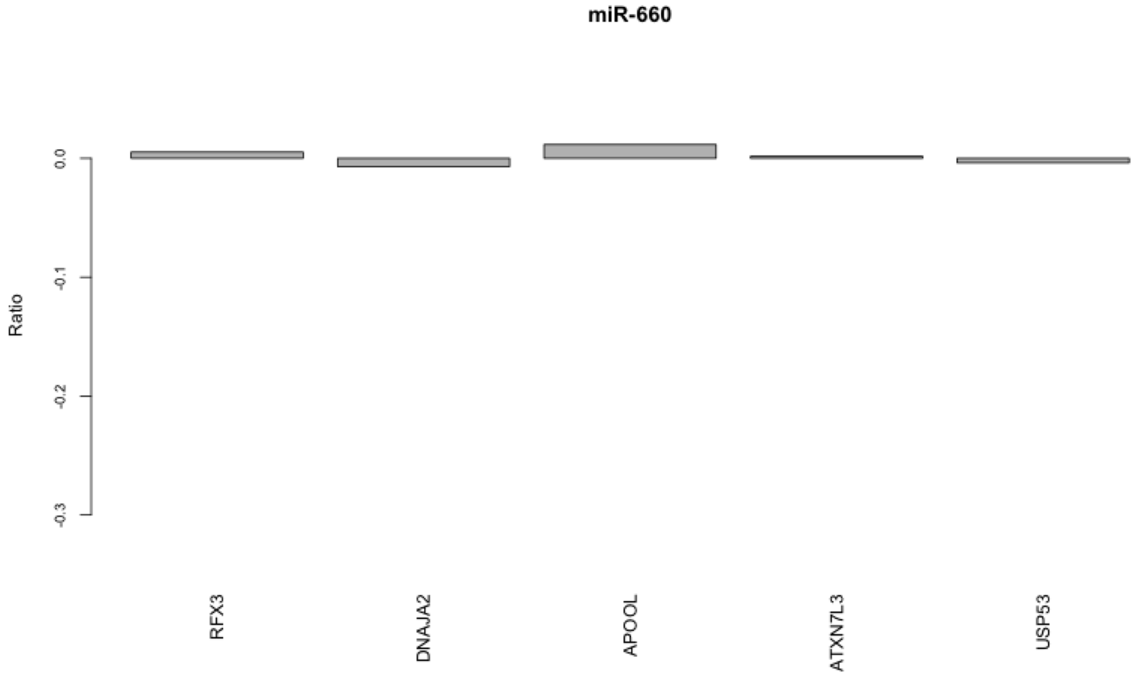

Supplementary Figure 50: Expression of the target genes of miR-660 in the CEPBA.dm groups compared to that of the rest.

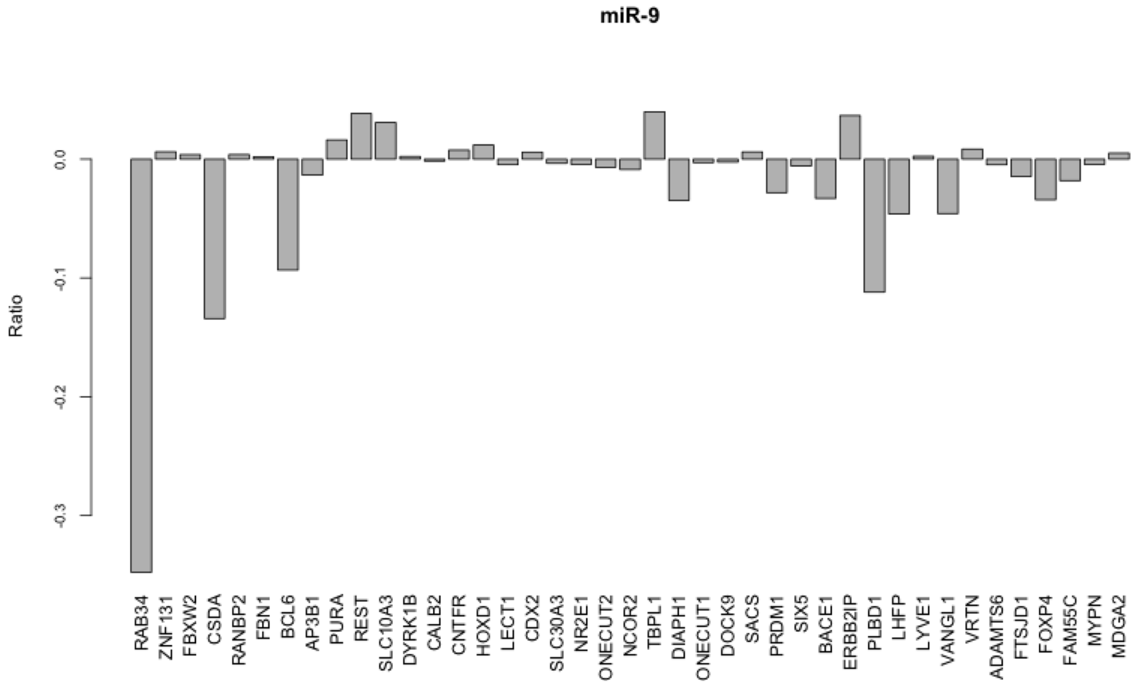

Supplementary Figure 51: Expression of the target genes of miR-9 in the CEPBA.dm groups compared to that of the rest.

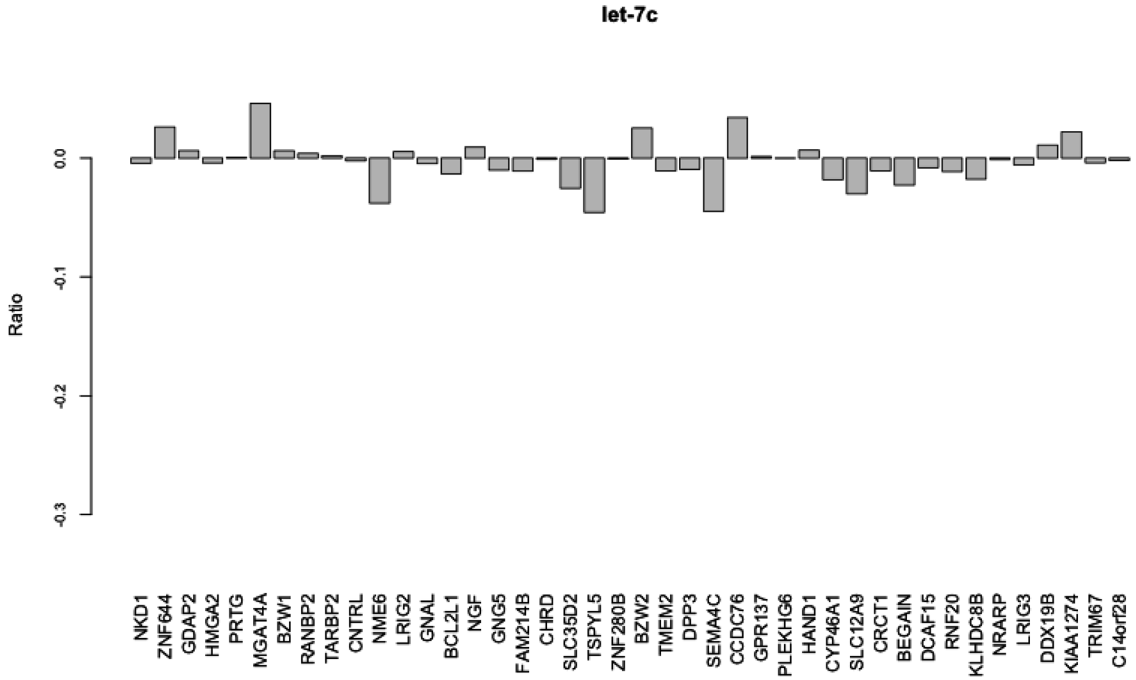

Supplementary Figure 52: Expression of the target genes of let-7c in the CEPBA.dm groups compared to that of the rest.

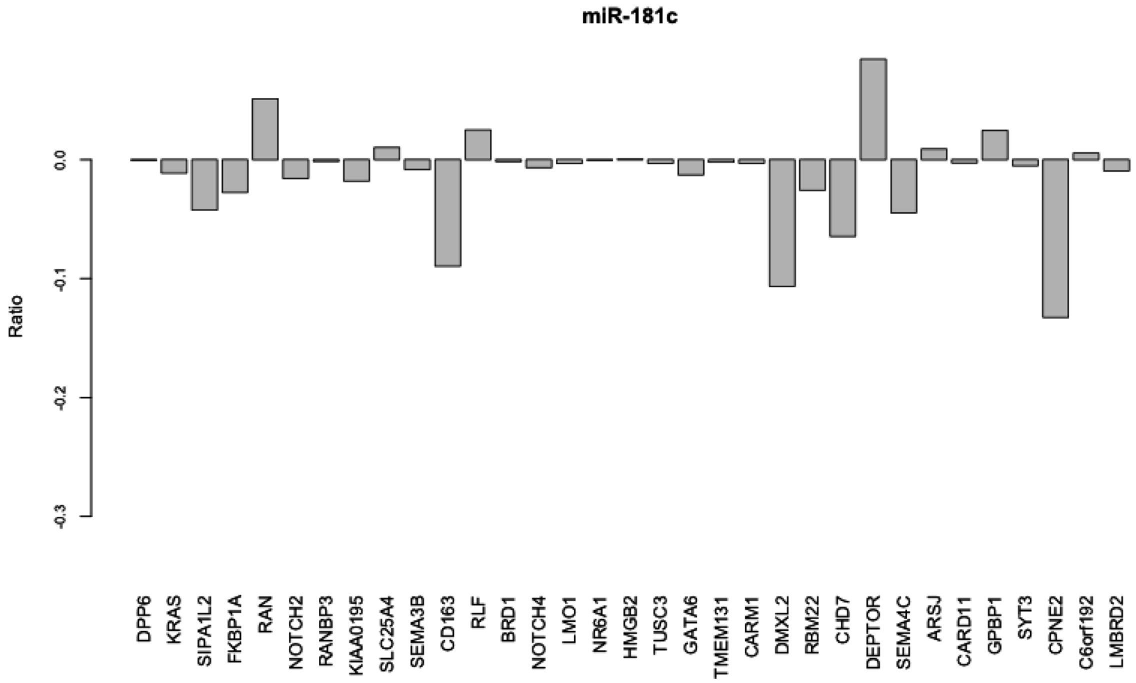

Supplementary Figure 53: Expression of the target genes of miR-181c in the CEPBA.dm groups compared to that of the rest.

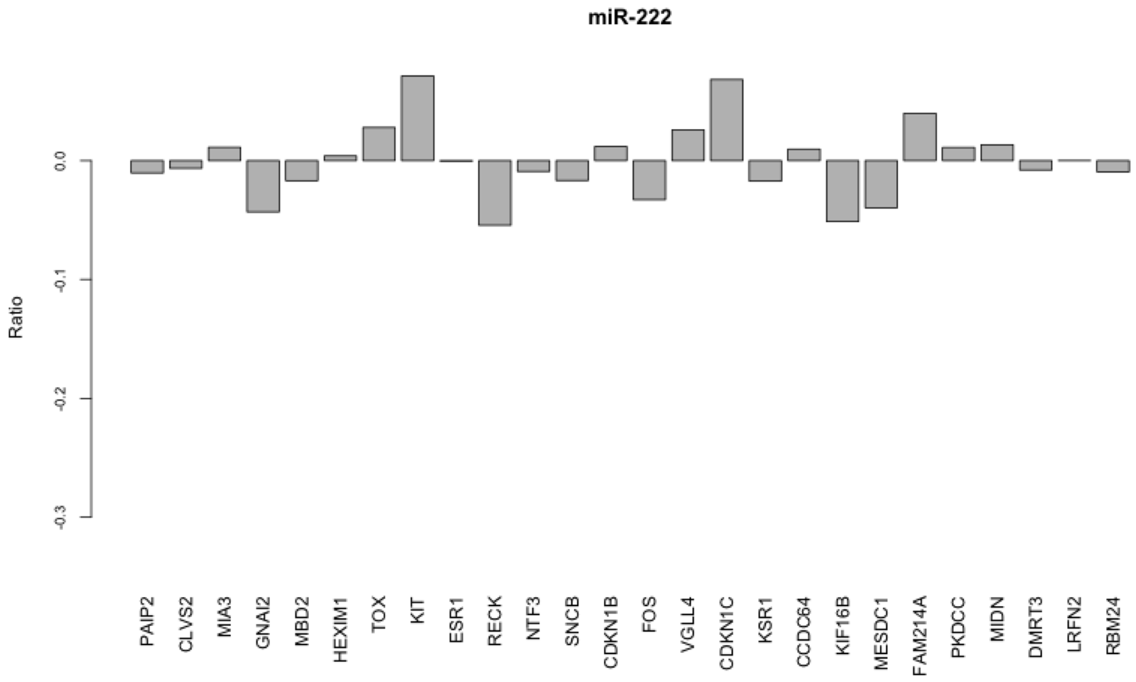

Supplementary Figure 54: Expression of the target genes of miR-222 in the CEPBA.dm groups compared to that of the rest.

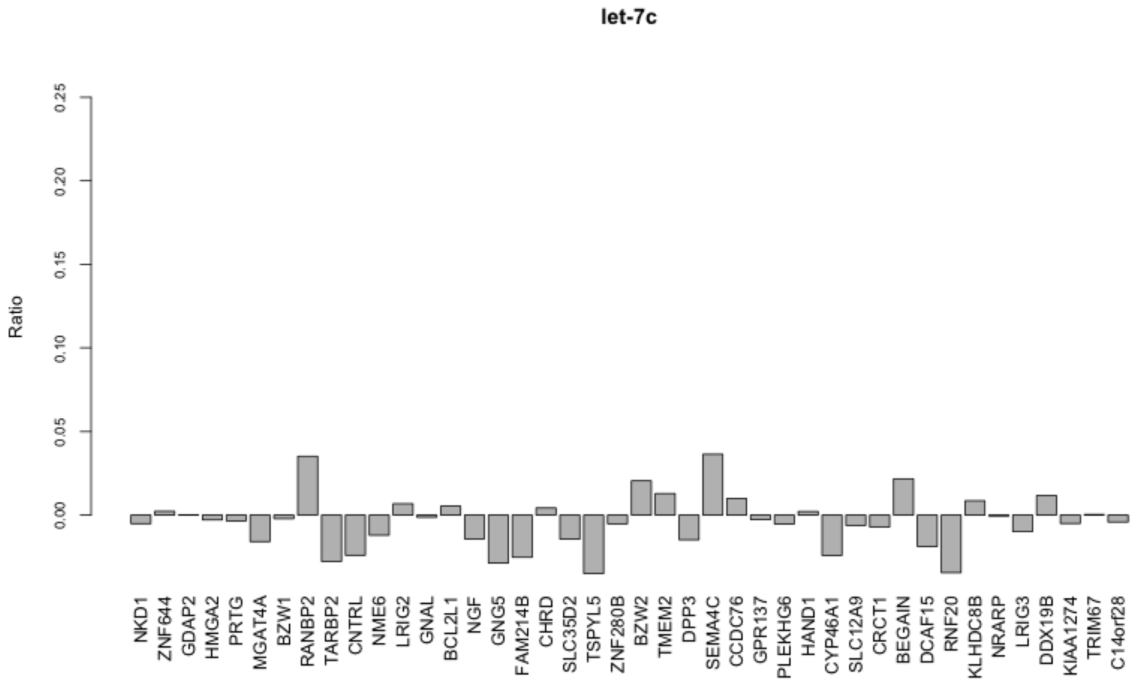

Supplementary Figure 55: Expression of the target genes of let-7c in the NPM1 groups compared to that of the rest.

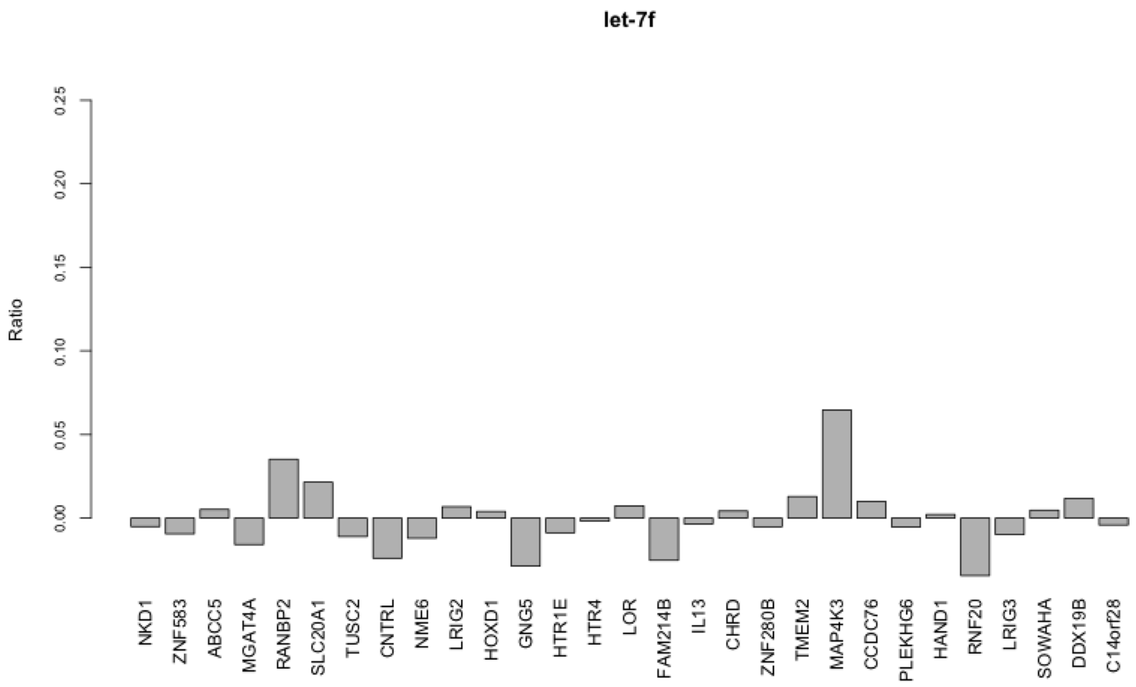

Supplementary Figure 56: Expression of the target genes of let-7f in the NPM1 groups compared to that of the rest.

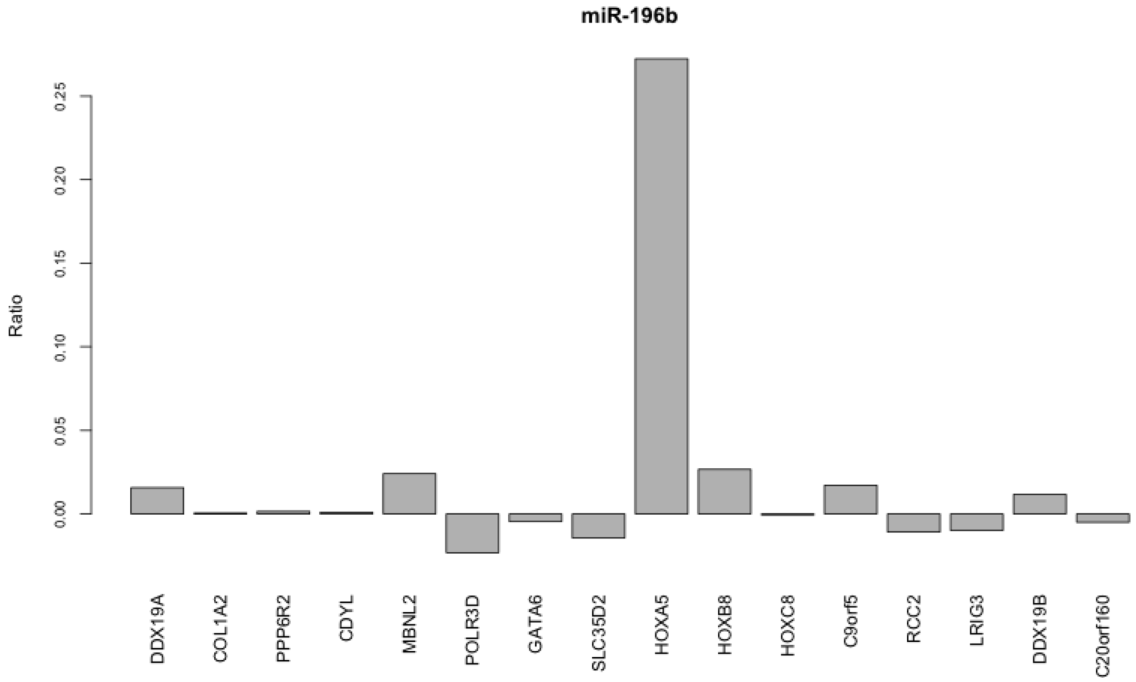

Supplementary Figure 57: Expression of the target genes of miR-196b in the NPM1 groups compared to that of the rest.

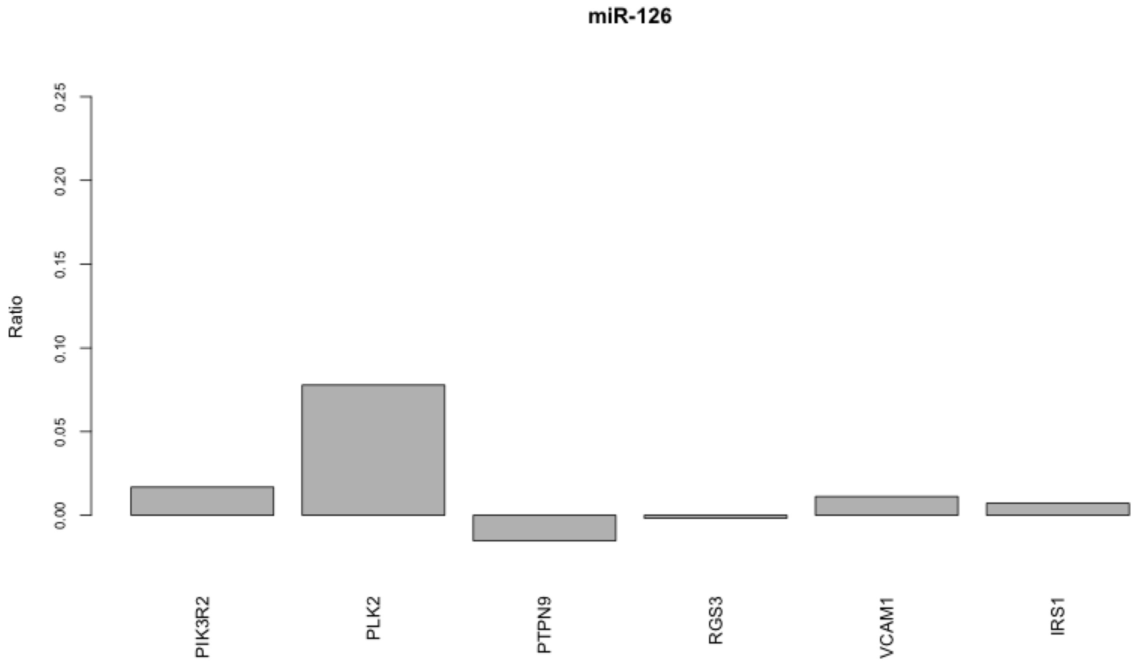

Supplementary Figure 58: Expression of the target genes of miR-126 in the NPM1 groups compared to that of the rest.

Supplementary Table 1: patient characteristics

**Gender**		***N***	**(%)**
	Male	86	(52.1)
	Female	79	(47.9)
**FAB**			
	M0	11	(6.7)
	M1	22	(13.3)
	M2	35	(21.1)
	M3	13	(7.9)
	M4	41	(24.8)
	M5	31	(18.8)
	M6	2	(1.2)
	M7	6	(3.6)
	Unknown	4	(2.4)
**Type 2**			
	MLL-rearrangement	36	(21.8)
	t(8;21)(q22;q22)	21	(12.7)
	inv(16)(p13q22)	17	(10.3)
	t(15;17)(q21;q22)	12	(7.3)
	t(7;12)(q36;p13)	5	(3.0)
	t(6;9)	4	(2.4)
	t(8;16)(p11;p13)	1	(0.6)
	MLL-PTD	2	(1.2)
	NUP98-NSD1	5	(3.0)
	NUP98-JARID1A	2	(1.2)
	NUP98-TOP1	1	(0.6)
	CEBPA homozygous	11	(6.7)
	NPM1	11	(6.7)
	None	35	(21.2)
	Other	2	(1.2)
		**Median**	**Range**
**Age** (years)		9.8	0.0–18.4
**WBC** (×10^9/L)		205	1–490

Supplementary Table 2: Characteristics per cluster. See Supplementary_Table_2

Supplementary Table 3: miRNA signatures were found in the five miRNA-mRNA target prediction databases we used in our analysis

MLL	t(8;21)	inv(16)	t(15;17)	CEPBA.dm	NPM1
let-7f	miR-126	let-7c	let-7f	let-7c	let-7c
miR-126	miR-130a	miR-126	miR-181c	miR-149	let-7f
miR-130a	miR-149	miR-130a	miR-195	miR-181c	miR-126
miR-133a	miR-196b	miR-203	miR-203	miR-196b	miR-196b
miR-149	miR-200c	miR-26a	miR-29a	miR-203	miR-320
miR-181c	miR-320	miR-30b	miR-369-3p	miR-222	
miR-195	miR-9	miR-30d	miR-409-5p	miR-363	
miR-196b		miR-335	miR-485-5p	miR-660	
miR-200c		miR-363		miR-9	
miR-203					
miR-222					
miR-26a					
miR-29a					
miR-30b					
miR-30d					
miR-335					
miR-363					
miR-660					
miR-9					

Supplementary Table 4: The miRNA signatures generated for each aberration group in the pediatric AML dataset. See Supplementary_Table_4

MATERIALS AND METHODS

Patient samples

Viably frozen diagnostic bone marrow or peripheral blood samples from a selection of 165 de novo pediatric AML cases were provided by the Dutch Childhood Oncology Group (DCOG), the AML 'Berlin-Frankfurt-Münster' Study Group (AML-BFM-SG), the Czech Pediatric Hematology Group (CPH) and the St. Louis Hospital in Paris, France. Samples were chosen to represent the most common and relevant cytogenetic groups, that is t(8;21)(q22;q22), inv(16)(p13q22), t(7;12)(q36;p13), and t(15;17)(q21;q22) as well as MLL-rearrangements and were selected based on the availability of high-quality RNA. Informed consent was obtained from all patients, after Institutional Review Board approval, according to national law and regulations and in accordance to the Declaration of Helsinki. Samples were enriched to contain at least 80% leukemic cells as previously described [1].

Cytogenetics and mutation analysis

Routine analysis of cytogenetic aberration was performed by standard chromosome banding analysis. In addition, the leukemic samples were screened for recurrent non-random cytogenetic aberrations characteristic for AML as described by the WHO classification 2008 [2] by RT-PCR and split-signal FISH as described previously [3]. Screening included MLL-rearrangements, inv(16)(p13q22), t(8;21)(q22;q22), t(15;17)(q21;q22), t(7;12)(q36;p13), t(8;16)(p11;p13), and NUP98-rearrangements as previously published [4, 5]. Samples were screened for hotspot mutations in NPM1, CEBPA, FLT3-ITD, NRAS, KRAS, PTPN11, KIT, and MLL-PTD as previously described [6–10].

MiRNA expression profiling

MicroRNA expression profiling was performed by Taqman® Array MicroRNA Cards v2.0 using Taqman technology in the 7900 HT Fast Real-Time PCR System (Applied Biosystems, Foster City, CA, USA). Raw Ct-values were analyzed, summarized and exported using SDS 2.3 (Applied Biosystems, Foster City, CA, USA). All further biostatistical analyses were performed using R 2.11.1 [11]. To measure expression of 665 miRs, two cards, A and B, were loaded with RNA from each sample. Per card type, Ct-values were imported (readCtData) into a qPCR-set using the package HTqPCR and correlation plots were called by the plotCtCor command [12]. The number of failed measurements (undetermined, Ct > 38, or flagged in any way by SDS software) per card type was summarized with the featureCategory command. Of this number the mean, standard deviation and 95% confidence interval of failed measurements were calculated to obtain the limits of the number of failed measurements. In the R environment, the median expression of each control per card was calculated. We calculated the mean value for all cards per control using these values. In addition, the standard deviation and 95% confidence interval were calculated to obtain the upper limits of expression. Cards were retained for further analyses if it was OK on visual inspection, the number of failed measurements was within 95% confidence interval of the median of failed measurements, and the value of the internal controls was within the 95% confidence interval of the group median for 2 out of 3 controls. If both card A and B for one sample were retained after going through the steps in our flowchart, expression values were merged into one dataset. Among the 66 miRNAs, the following nine were not expressed in any sample: miR-384, miR-509-5p, miR-516a-5p, miR-887, miR-891b, miR-892a, miR-30c, miR-519e, miR-608. They were excluded from downstream analysis.

Statistical analyses

Unsupervised clustering

The normalized expression of 664 miRs underwent a filtering in order to make each value in the dataset strictly positive. Shortly; 26 was added to each miR expression value, successively the geometric mean expression was calculated for each miR. Hierarchical clustering of samples was performed on the subset of 563 miRs that had 6-fold increase or decrease expressions compared to the geometric mean in at least one sample with Pearson correlation and Ward distance. The 6-fold change cut-off was chosen as this resulted in the most coherent clustering pattern with a limited number of clusters when plots were made with cut-offs ranging from 2- to 16-fold change. The clustering result was visualized using Heatmapper [13]. In addition, correlation between miRs was calculated, and visualized using the R-package gplots [14]. The clustering was performed within the R 3.0.3 statistical environment.

Classifying pediatric AML- selection of miRs and construction of classifier

We used the pediatric AML dataset to generate miRNA signatures specific to each cytogenetic subtype (i.e., via 1-vs.-Rest multiclass classification scheme) using Support Vector Machines (SVM). Our strategy to extract both signatures is similar to the one used in Balgobindet al. (2011). To obtain reliable results, we performed 100 outer-cross-validation (OCV) and 100 inner-cross-validation (ICV) on the dataset. In each of 100 OCV iterations the following steps were taken: the dataset was randomly divided into a training set (4/5 samples) and a test set (1/5 samples). Then, 100 ICV iterations were performed on the training set. In each of 100 ICV iterations the training set was further divided, again subject to stratification constraints, into an inner-training (2/3) and an inner-test (1/3) set. We applied an empirical Bayes linear regression model on the inner-training set to select top 50 miRNAs

that discriminate each AML subtype under consideration from the rest.

The selected features (50 miRNAs) were processed in following ways to obtain reduced set of miRNA signatures. For each subtype, the miRNA signatures were ranked (from best to worst) according to their association with the subtype via the global test. Subsequently, an SVM classifier was trained (e.g. MLL vs. Rest) using the 50 miRNAs and predictive performance on the inner-test set was gauged. The size of the signature list was then reduced from 50 by removing the miRNA located at the bottom of the list, and the classification was re-run. This process was repeated until there were no more than 5 genes remaining and mean sensitivity (across subtypes) was recorded. Finally, the list that corresponds to the median sensitivity over the 3 ICV train-test procedures is chosen.

The average classification for each subtype, which is calculated by using 100 ICV iterations, is reported. Balgobind et al. (2011) argued that this double-loop CV avoids over-fitting and leads to stable signatures with highest prediction accuracy.

A signature list of smallest size that renders highest prediction sensitivity was determined and a classifier is trained using these miRNA signatures only on the pediatric AML dataset. Then, the trained classifier was applied to the adult AML dataset to predict subtype labels.

To reliably estimate the discriminator power of miRNA data included in this study, multiple well-known classification algorithms were utilized to create an ideal test bed. Beside the SVM algorithm mentioned in the paper, we used two other well-known classification algorithms: Random Forest (RF) and prediction analysis for microarrays (PAM). Classifications were performed using the strategy mentioned in the paper. Classification accuracies in different cytogenetic subtypes from three different algorithms are shown in Supplementary Figures 2 and 3.

Classifying miRNA signatures are characterized by distinct target gene patterns

To examine if the miRNA signatures given in Table 3 are characterized by distinct target gene patterns, thus reflecting disease biology, we performed the following analyses: we downloaded 289 miRNAs from five miRNA-mRNA target prediction databases: microcosm, mirecords, mirtarbase and pita targetscan. Among the 47 unique miRNAs given in Table 3, only 25 were found in the miRNA-mRNA target prediction databases we downloaded (See Supplementary Table 3). To reliably predict miRNA target genes, we call a gene as the target of the miRNA under investigation if the prediction is reported at least in three databases.

It is known that a miRNA expression is inversely correlated with the expression of its targeted genes. To investigate if this prior knowledge holds in our dataset, we obtained mRNA expression dataset that were measured on the same samples (Balgobind et al 2011). Visualizations of the inverse correlation between miRNA and targeted genes were realized via the following steps: (1) targeted genes of the signature miRNA were identified in the mRNA expression dataset; (2) The aberration group to which the miRNA under investigation was called signature is noted, and ratio of the mean expression of each target gene in the aberration group and the mean expression in the rest was calculated. For example, say a miRNA is signature of the MLL group, and it has multiple targeted genes. For each gene we calculate the following:

$$Ratio_i = \log_2\left(\frac{m_i^{MLL}}{m_i^{Rest}}\right)$$

where m_i^{MLL} is the mean expression of the ith gene calculated using samples from the MLL group. m_i^{Rest} is the mean expression of the same gene calculated using samples from the non-MLL (rest) samples. We took log2 of the ratio for better visualization purpose; (3) the calculated ratios of the targeted genes were visualized using boxpot. If a miRNA specific to the MLL group has lower expression in that group than that of the rest, we expect (most of) its targeted genes have *Ratio* of larger than 1.

Supplementary Figures 4–58 display the expression of genes predicted to be target of the signature miRNAs.

REFERENCES

1. Kaspers GJ, Veerman AJ, Pieters R, Broekema GJ, Huismans DR, Kazemier KM, Loonen AH, Rottier MA, van Zantwijk CH, Hählen K, Wering ER. Mononuclear cells contaminating acute lymphoblastic leukaemic samples tested for cellular drug resistance using the methyl-thiazol-tetrazolium assay. Br J Cancer. 1994; 70:1047–1052.
2. Vardiman JW, Thiele J, Arber DA, Brunning RD, Borowitz MJ, Porwit A, Harris NL, Le Beau MM, Hellström-Lindberg E, Tefferi A, Bloomfield CD. The 2008 revision of the World Health Organization (WHO) classification of myeloid neoplasms and acute leukemia: rationale and important changes. Blood. 2009; 114:937–951.
3. Balgobind BV, Van den Heuvel-Eibrink MM, De Menezes RX, Reinhardt D, Hollink IH, Arentsen-Peters ST, van Wering ER, Kaspers GJ, Cloos J, de Bont ES, Cayuela JM, Baruchel A, Meyer C, et al. Evaluation of gene expression signatures predictive of cytogenetic and molecular subtypes of pediatric acute myeloid leukemia. Haematologica. 2011; 96:221–230.
4. Coenen EA, Zwaan CM, Reinhardt D, Harrison CJ, Haas OA, de Haas V, Mihál V, De Moerloose B, Jeison M, Rubnitz JE, Tomizawa D, Johnston D, Alonzo TA, et al. Pediatric acute myeloid leukemia with t(8;16)(p11;p13), a distinct clinical and biological entity: a collaborative study by the International-Berlin-Frankfurt-Munster AML-study group. Blood. 2013; 122:2704–2713.
5. Hollink IH, van den Heuvel-Eibrink MM, Arentsen-Peters ST, Pratcorona M, Abbas S, Kuipers JE, van Galen JF, Beverloo HB, Sonneveld E, Kaspers GJ,

Trka J, Baruchel A, Zimmermann M, et al. NUP98/NSD1 characterizes a novel poor prognostic group in acute myeloid leukemia with a distinct HOX gene expression pattern. Blood. 2011; 118:3645–3656.

6. Balgobind BV, Van Vlierberghe P, van den Ouweland AM, Beverloo HB, Terlouw-Kromosoeto JN, van Wering ER, Reinhardt D, Horstmann M, Kaspers GJ, Pieters R, Zwaan CM, Van den Heuvel-Eibrink MM, Meijerink JP. Leukemia-associated NF1 inactivation in patients with pediatric T-ALL and AML lacking evidence for neurofibromatosis. Blood. 2008; 111:4322–4328.

7. Barjesteh van Waalwijk van Doorn-Khosrovani S, Erpelinck C, Meijer J, van Oosterhoud S, van Putten WL, Valk PJ, Berna Beverloo H, Tenen DG, Löwenberg B, Delwel R. Biallelic mutations in the CEBPA gene and low CEBPA expression levels as prognostic markers in intermediate-risk AML. Hematol J. 2003; 4:31–40.

8. Kiyoi H, Naoe T, Yokota S, Nakao M, Minami S, Kuriyama K, Takeshita A, Saito K, Hasegawa S, Shimodaira S, Tamura J, Shimazaki C, Matsue K, et al. Internal tandem duplication of FLT3 associated with leukocytosis in acute promyelocytic leukemia. Leukemia Study Group of the Ministry of Health and Welfare (Kohseisho). Leukemia. 1997; 11:1447–1452.

9. Yamamoto Y, Kiyoi H, Nakano Y, Suzuki R, Kodera Y, Miyawaki S, Asou N, Kuriyama K, Yagasaki F, Shimazaki C, Akiyama H, Saito K, Nishimura M, et al. Activating mutation of D835 within the activation loop of FLT3 in human hematologic malignancies. Blood. 2001; 97:2434–2439.

10. Caligiuri MA, Strout MP, Schichman SA, Mrózek K, Arthur DC, Herzig GP, Baer MR, Schiffer CA, Heinonen K, Knuutila S, Nousiainen T, Ruutu T, Block AW, et al. Partial tandem duplication of ALL1 as a recurrent molecular defect in acute myeloid leukemia with trisomy 11. Cancer Res. 1996; 56:1418–1425.

11. Team RC. R: A Language and Environment for Statistical Computing. R Foundation for Statistical Computing, 2015.

12. Dvinge H, Bertone P. HTqPCR: high-throughput analysis and visualization of quantitative real-time PCR data in R. Bioinformatics. 2009; 25:3325–3326.

13. Verhaak RG, Sanders MA, Bijl MA, Delwel R, Horsman S, Moorhouse MJ, van der Spek PJ, Löwenberg B, Valk PJ. HeatMapper: powerful combined visualization of gene expression profile correlations, genotypes, phenotypes and sample characteristics. BMC Bioinformatics. 2006; 7:337.

14. Warnes GR BB, Bonebakker L, Gentleman R, Liaw WHA, Lumley T, Maechler M, Magnusson A, Moeller S, Schwartz M, Venables B. gplots: Various R programming tools for plotting data. R package version 2.12.1 ed, 2013.
