# Supplementary material for: Classification of pediatric acute myeloid leukemia based on miRNA expression profiles
Source: Oncotarget. 2017 Mar 23;8(20):33078–85. doi: 10.18632/oncotarget.16525 (PMC5464851; doi:10.18632/oncotarget.16525)
Supplement: Supplementary file 3 [file oncotarget-08-33078-s003.docx]

Supplementary Table 4: The miRNA signatures generated for each aberration group in the pediatric AML dataset. The number (1-6) in the second column indicates which group a miRNA specific to. The value 1-6 denote *MLL*-rearrangements, t(8;21), inv(16), t(15;17), *CEPBA*.dm and *NPM1* groups, respectively. The values in columns 3-8 indicate the miRNA mean expression. The mean expression of a miRNA in a group is calculated by using the samples belong to that group only.

| **miRNA** | **Group** | **MLL** | **t(8;21)** | **inv(16)** | **t(15;17)** | **CEPBA.dm** | **NPM1** |
| --- | --- | --- | --- | --- | --- | --- | --- |
| let-7f | 1 | -7.653 | -7.876 | -7.041 | -5.746 | -7.323 | -6.916 |
| miR-126 | 1 | -5.856 | -5.988 | -5.838 | -5.346 | -6.234 | -4.207 |
| miR-126* | 1 | -13.607 | -14.476 | -9.869 | -15.014 | -10.775 | -11.462 |
| miR-130a | 1 | -7.530 | -7.771 | -6.963 | -5.804 | -7.173 | -7.589 |
| miR-133a | 1 | -4.660 | -4.587 | -3.959 | -2.686 | -4.397 | -3.796 |
| miR-139-5p | 1 | -9.759 | -9.405 | -8.416 | -7.177 | -8.648 | -9.132 |
| miR-181a | 1 | -15.063 | -18.434 | -13.140 | -17.581 | -21.048 | -11.263 |
| miR-181a* | 1 | -10.324 | -12.129 | -8.950 | -3.967 | -9.978 | -8.654 |
| miR-181c | 1 | -10.796 | -10.880 | -9.979 | -9.412 | -9.683 | -9.895 |
| miR-195 | 1 | -7.560 | -8.040 | -7.408 | -6.549 | -7.454 | -8.097 |
| miR-199a-3p | 1 | -0.732 | -1.253 | -0.705 | -0.039 | -0.915 | -0.357 |
| miR-203 | 1 | -13.434 | -15.222 | -12.236 | -15.017 | -14.626 | -14.492 |
| miR-222 | 1 | -13.596 | -16.373 | -11.667 | -10.864 | -17.142 | -5.284 |
| miR-26a | 1 | -20.814 | -21.883 | -20.252 | -24.070 | -22.824 | -6.342 |
| miR-26a-1* | 1 | -24.180 | -21.938 | -25.000 | -25.000 | -23.971 | -24.105 |
| miR-29a | 1 | -22.479 | -18.644 | -17.327 | -19.347 | -22.870 | -19.928 |
| miR-30b | 1 | -10.867 | -7.810 | -7.526 | -6.847 | -13.640 | -9.435 |
| miR-30d | 1 | -12.097 | -14.654 | -8.653 | -6.061 | -9.291 | -10.487 |
| miR-335 | 1 | -21.345 | -18.246 | -19.630 | -5.801 | -18.924 | -19.870 |
| miR-340-5p | 1 | -21.440 | -23.937 | -23.877 | -22.676 | -25.000 | -25.000 |
| miR-374b | 1 | -9.102 | -9.560 | -9.201 | -7.890 | -10.339 | -9.469 |
| miR-625 | 1 | -20.896 | -21.646 | -21.224 | -15.985 | -20.429 | -22.982 |
| miR-766 | 1 | -17.810 | -9.675 | -9.147 | -18.351 | -17.438 | -11.908 |
| miR-149 | 1 | -4.795 | -4.450 | -4.479 | -3.187 | -4.002 | -4.255 |
| miR-196b | 1 | -23.946 | -22.547 | -25.000 | -24.368 | -24.297 | -23.828 |
| miR-200c | 1 | -5.056 | -5.160 | -4.787 | -4.066 | -4.656 | -4.952 |
| miR-362-5p | 1 | -11.398 | -10.804 | -11.132 | -9.044 | -9.066 | -11.461 |
| miR-363 | 1 | -22.143 | -21.859 | -21.962 | -21.367 | -21.235 | -20.259 |
| miR-500* | 1 | -22.797 | -21.774 | -22.321 | -9.520 | -21.135 | -22.150 |
| miR-500a | 1 | -18.805 | -20.216 | -16.154 | -19.705 | -22.565 | -13.580 |
| miR-502-3p | 1 | -21.604 | -18.348 | -16.497 | -19.920 | -13.493 | -14.290 |
| miR-582-5p | 1 | -19.040 | -16.980 | -15.315 | -17.055 | -12.009 | -15.559 |
| miR-660 | 1 | -24.070 | -18.519 | -16.855 | -21.774 | -23.777 | -22.089 |
| miR-885-5p | 1 | -8.350 | -8.395 | -8.048 | -8.248 | -8.395 | -8.065 |
| miR-9 | 1 | -4.344 | -4.923 | -4.333 | -4.405 | -4.474 | -4.222 |
| miR-9* | 1 | -14.601 | -17.079 | -17.031 | -14.900 | -14.239 | -15.052 |
| miR-944 | 1 | -1.912 | -1.576 | -1.521 | -1.753 | -1.764 | -1.629 |
| miR-149 | 2 | -4.795 | -4.450 | -4.479 | -3.187 | -4.002 | -4.255 |
| miR-196b | 2 | -23.946 | -22.547 | -25.000 | -24.368 | -24.297 | -23.828 |
| miR-200c | 2 | -5.056 | -5.160 | -4.787 | -4.066 | -4.656 | -4.952 |
| miR-27a* | 2 | -9.342 | -10.280 | -9.093 | -8.466 | -9.409 | -9.032 |
| miR-582-5p | 2 | -19.040 | -16.980 | -15.315 | -17.055 | -12.009 | -15.559 |
| miR-885-5p | 2 | -8.350 | -8.395 | -8.048 | -8.248 | -8.395 | -8.065 |
| miR-9 | 2 | -4.344 | -4.923 | -4.333 | -4.405 | -4.474 | -4.222 |
| miR-9* | 2 | -14.601 | -17.079 | -17.031 | -14.900 | -14.239 | -15.052 |
| miR-126 | 2 | -5.856 | -5.988 | -5.838 | -5.346 | -6.234 | -4.207 |
| miR-126* | 2 | -13.607 | -14.476 | -9.869 | -15.014 | -10.775 | -11.462 |
| miR-130a | 2 | -7.530 | -7.771 | -6.963 | -5.804 | -7.173 | -7.589 |
| miR-139-5p | 2 | -9.759 | -9.405 | -8.416 | -7.177 | -8.648 | -9.132 |
| miR-26a-1* | 2 | -24.180 | -21.938 | -25.000 | -25.000 | -23.971 | -24.105 |
| miR-320 | 2 | -6.518 | 1.026 | 0.487 | -3.534 | -1.874 | -3.314 |
| let-7c | 3 | -18.306 | -16.894 | -17.946 | -17.890 | -17.230 | -16.599 |
| miR-126 | 3 | -5.856 | -5.988 | -5.838 | -5.346 | -6.234 | -4.207 |
| miR-126* | 3 | -13.607 | -14.476 | -9.869 | -15.014 | -10.775 | -11.462 |
| miR-130a | 3 | -7.530 | -7.771 | -6.963 | -5.804 | -7.173 | -7.589 |
| miR-139-5p | 3 | -9.759 | -9.405 | -8.416 | -7.177 | -8.648 | -9.132 |
| miR-199a-3p | 3 | -0.732 | -1.253 | -0.705 | -0.039 | -0.915 | -0.357 |
| miR-203 | 3 | -13.434 | -15.222 | -12.236 | -15.017 | -14.626 | -14.492 |
| miR-26a | 3 | -20.814 | -21.883 | -20.252 | -24.070 | -22.824 | -6.342 |
| miR-26a-1* | 3 | -24.180 | -21.938 | -25.000 | -25.000 | -23.971 | -24.105 |
| miR-27a* | 3 | -9.342 | -10.280 | -9.093 | -8.466 | -9.409 | -9.032 |
| miR-30b | 3 | -10.867 | -7.810 | -7.526 | -6.847 | -13.640 | -9.435 |
| miR-30d | 3 | -12.097 | -14.654 | -8.653 | -6.061 | -9.291 | -10.487 |
| miR-335 | 3 | -21.345 | -18.246 | -19.630 | -5.801 | -18.924 | -19.870 |
| miR-766 | 3 | -17.810 | -9.675 | -9.147 | -18.351 | -17.438 | -11.908 |
| miR-944 | 3 | -1.912 | -1.576 | -1.521 | -1.753 | -1.764 | -1.629 |
| miR-363 | 3 | -22.143 | -21.859 | -21.962 | -21.367 | -21.235 | -20.259 |
| miR-500a | 3 | -18.805 | -20.216 | -16.154 | -19.705 | -22.565 | -13.580 |
| let-7f | 4 | -7.653 | -7.876 | -7.041 | -5.746 | -7.323 | -6.916 |
| miR-181c | 4 | -10.796 | -10.880 | -9.979 | -9.412 | -9.683 | -9.895 |
| miR-195 | 4 | -7.560 | -8.040 | -7.408 | -6.549 | -7.454 | -8.097 |
| miR-199a-3p | 4 | -0.732 | -1.253 | -0.705 | -0.039 | -0.915 | -0.357 |
| miR-203 | 4 | -13.434 | -15.222 | -12.236 | -15.017 | -14.626 | -14.492 |
| miR-29a | 4 | -22.479 | -18.644 | -17.327 | -19.347 | -22.870 | -19.928 |
| miR-369-3p | 4 | -22.841 | -23.869 | -25.000 | -22.925 | -23.571 | -25.000 |
| miR-369-5p | 4 | -12.282 | -7.503 | -7.682 | -7.099 | -9.085 | -9.940 |
| miR-374b | 4 | -9.102 | -9.560 | -9.201 | -7.890 | -10.339 | -9.469 |
| miR-409-5p | 4 | -8.209 | -8.925 | -7.853 | -8.816 | -7.991 | -7.766 |
| miR-485-5p | 4 | -15.148 | -13.305 | -9.973 | -13.058 | -12.280 | -8.869 |
| miR-625 | 4 | -20.896 | -21.646 | -21.224 | -15.985 | -20.429 | -22.982 |
| miR-654-3p | 4 | -23.169 | -21.816 | -25.000 | -23.667 | -21.838 | -23.296 |
| miR-654-5p | 4 | -12.591 | -12.893 | -14.249 | -15.383 | -18.662 | -16.161 |
| miR-139-5p | 5 | -9.759 | -9.405 | -8.416 | -7.177 | -8.648 | -9.132 |
| miR-149 | 5 | -4.795 | -4.450 | -4.479 | -3.187 | -4.002 | -4.255 |
| miR-196b | 5 | -23.946 | -22.547 | -25.000 | -24.368 | -24.297 | -23.828 |
| miR-203 | 5 | -13.434 | -15.222 | -12.236 | -15.017 | -14.626 | -14.492 |
| miR-26a-1* | 5 | -24.180 | -21.938 | -25.000 | -25.000 | -23.971 | -24.105 |
| miR-27a* | 5 | -9.342 | -10.280 | -9.093 | -8.466 | -9.409 | -9.032 |
| miR-363 | 5 | -22.143 | -21.859 | -21.962 | -21.367 | -21.235 | -20.259 |
| miR-500a | 5 | -18.805 | -20.216 | -16.154 | -19.705 | -22.565 | -13.580 |
| miR-502-3p | 5 | -21.604 | -18.348 | -16.497 | -19.920 | -13.493 | -14.290 |
| miR-660 | 5 | -24.070 | -18.519 | -16.855 | -21.774 | -23.777 | -22.089 |
| miR-9 | 5 | -4.344 | -4.923 | -4.333 | -4.405 | -4.474 | -4.222 |
| miR-9* | 5 | -14.601 | -17.079 | -17.031 | -14.900 | -14.239 | -15.052 |
| let-7c | 5 | -18.306 | -16.894 | -17.946 | -17.890 | -17.230 | -16.599 |
| miR-181a | 5 | -15.063 | -18.434 | -13.140 | -17.581 | -21.048 | -11.263 |
| miR-181a* | 5 | -10.324 | -12.129 | -8.950 | -3.967 | -9.978 | -8.654 |
| miR-181c | 5 | -10.796 | -10.880 | -9.979 | -9.412 | -9.683 | -9.895 |
| miR-222 | 5 | -13.596 | -16.373 | -11.667 | -10.864 | -17.142 | -5.284 |
| miR-340-5p | 5 | -21.440 | -23.937 | -23.877 | -22.676 | -25.000 | -25.000 |
| let-7c | 6 | -18.306 | -16.894 | -17.946 | -17.890 | -17.230 | -16.599 |
| let-7f | 6 | -7.653 | -7.876 | -7.041 | -5.746 | -7.323 | -6.916 |
| miR-196b | 6 | -23.946 | -22.547 | -25.000 | -24.368 | -24.297 | -23.828 |
| miR-320 | 6 | -6.518 | 1.026 | 0.487 | -3.534 | -1.874 | -3.314 |
| miR-126 | 6 | -5.856 | -5.988 | -5.838 | -5.346 | -6.234 | -4.207 |

Note: mRNA names in red denote they were upregulated (on average) in that particular

group compared the rest. Where as, mRNAs in green denote they were downregulated.
